# Neuroblastoma Origin and Therapeutic Targets for Immunotherapy

**DOI:** 10.1155/2018/7394268

**Published:** 2018-07-11

**Authors:** Irina V. Kholodenko, Daniel V. Kalinovsky, Igor I. Doronin, Sergey M. Deyev, Roman V. Kholodenko

**Affiliations:** ^1^Orekhovich Institute of Biomedical Chemistry, 10 Pogodinskaya St., Moscow 119121, Russia; ^2^Shemyakin-Ovchinnikov Institute of Bioorganic Chemistry, Russian Academy of Sciences, 16/10 Miklukho-Maklaya St., Moscow 117997, Russia; ^3^Real Target LLC, 16/10 Miklukho-Maklaya St., Moscow 117997, Russia; ^4^Institute of Engineering Physics for Biomedicine (PhysBio), National Research Nuclear University “MEPhI”, Moscow 115409, Russia

## Abstract

Neuroblastoma is a pediatric solid cancer of heterogeneous clinical behavior. The unique features of this type of cancer frequently hamper the process of determining clinical presentation and predicting therapy effectiveness. The tumor can spontaneously regress without treatment or actively develop and give rise to metastases despite aggressive multimodal therapy. In recent years, immunotherapy has become one of the most promising approaches to the treatment of neuroblastoma. Still, only one drug for targeted immunotherapy of neuroblastoma, chimeric monoclonal GD2-specific antibodies, is used in the clinic today, and its application has significant limitations. In this regard, the development of effective and safe GD2-targeted immunotherapies and analysis of other potential molecular targets for the treatment of neuroblastoma represents an important and topical task. The review summarizes biological characteristics of the origin and development of neuroblastoma and outlines molecular markers of neuroblastoma and modern immunotherapy approaches directed towards these markers.

## 1. Introduction

Neuroblastoma (NB) is the most common extracranial solid tumor in children, accounting for 7% of all pediatric neoplasms in patients under 15 years and 15% of all pediatric deaths caused by cancers. It is the second most common type of pediatric solid tumors surpassed only by CNS tumors and comes third after leukemia and brain tumors in terms of incidence rates among pediatric cancers. World mortality rates are 0.85–1.1 cases per 100,000 children under the age of 15 [1].

Neuroblastoma is a complex type of tumor with unique features. The biological heterogeneity of neuroblastoma results in a variety of clinical presentations of this cancer. In some patients, neuroblastoma may completely regress or spontaneously differentiate, which leads to complete recovery without any treatment. In other cases, children with neuroblastoma develop a widespread metastatic tumor with very poor outcomes [2].

Despite the fact that mass screening of neuroblastoma does not significantly improve outcome for patients [3], some success in NB therapy has been achieved in recent years, primarily due to introduction of novel therapeutic approaches. Patients with low- and intermediate-risk neuroblastoma have favorable prognosis and an excellent five-year survival rate of more than 90%. However, in the case of high-risk neuroblastoma (HR-NB), which is detected in approximately 60% of cases, the prognosis of treatment remains unfavorable. Despite aggressive multimodal therapy, the five-year survival rate remains under 50% [2]. The standard methods of neuroblastoma therapy have strong side effects, including serious damage to internal organs, anemia, effects on fertility, and hair loss. Chemotherapy, radiotherapy, and surgical methods demonstrate particularly low efficacy on the late stages of the disease treatment; they also do not solve the problem of minimal residual disease which is the cause of subsequent relapse.

The reasons for the low effectiveness of HR-NB therapy by standard methods lie in the biological and immunological features of this cancer. Neuroblastoma evades the control of the immune system and manifests high cell heterogeneity, considerably limiting the efficacy of currently used approaches such as high-dose chemotherapy, surgery, and radiotherapy.

Immunotherapy represents a promising approach in the treatment of HR-NB. Currently, monoclonal GD2-specific antibodies are approved for the treatment of HR-NB in combination therapy. The use of GD2-specific antibodies significantly increases the survival of patients [4] and is becoming the standard approach of therapy for this type of cancer [5]. At the same time, the use of this immunotherapeutic approach cannot be considered optimal because of the significant side effects that limit the dose intensity of the drug and the effectiveness of therapy in general. Still, administration of monoclonal antibodies does not result in cumulative or long-term toxicity, and, therefore, immunotherapy remains an attractive approach for HR-NB treatment. In this regard, a deep understanding of the biological features of NB, search and analysis of molecular markers on neuroblastoma cells, and adaptation of modern immunotherapeutic approaches for the treatment of HR-NB are important milestones for developing effective neuroblastoma immunotherapy.

## 2. Origin of Neuroblastoma

**Figure 1:**
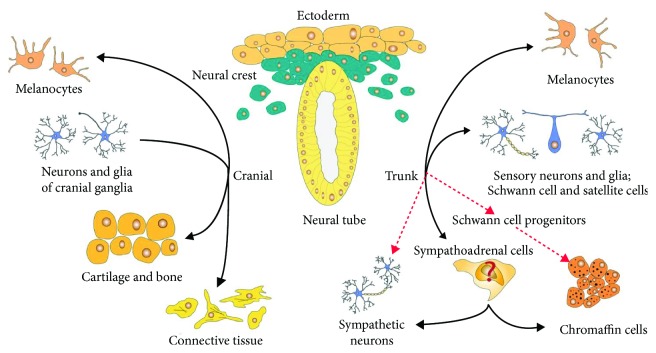
Cell types of neural crest origin.

Neural crest cells are a population of cells found only in vertebrates. The neural crest arises from the embryonic ectoderm and develops from the neural tube after its closure [6]. The differentiation of neural crest cells into a wide range of cell types contributes to the emergence of diverse anatomical structures and occurs due to the epithelial-to-mesenchymal transition (EMT), a process by which cells lose polarity and gain reduced adhesion, which allows the neural crest cells to delaminate and migrate from the neural tube. These cells individually or collectively migrate along stereotyped paths and reach numerous, often remote parts of the embryo, where eventually they differentiate into a diverse array of cell types, including melanocytes, craniofacial cartilage cells and bones, smooth muscle cells, peripheral neurons, and glial cells [7]. A complex of epigenetic and transcriptional programs regulates the delamination, migration, and postmigratory differentiation of neural crest cells. These programs include histone modification, DNA methylation, and expression of bone morphogenetic proteins and transcription factors [8]. Neural crest cells can be divided into five functional types: vagal, sacral, cranial, cardiac, and trunk cells. Thus, the neural crest cells represent a transitional type of cells that quickly passes from multipotent progenitors to a variety of differentiated cell types, from neurons and glia of the peripheral nervous system to melanocytes, cartilage, and bone cells of the craniofacial skeleton [7]. The main cell types of neural crest origin are presented on Figure 1.

It was originally considered that neural crest cells gradually lose their multipotent properties and/or plasticity when they reach the postmigration stage. To date, it has been proved that some subpopulations with high plasticity and the ability to form spheres *in vitro* are retained in various tissues at late embryonic and postnatal stages, and even in the adult body [9]. In the adult organism, progenitor cells of the neural crest are found in many types of tissues, including skin [10, 11], dorsal root ganglia [12], adrenal medulla [13], bone marrow [14], and a number of other tissues [15]. It has been shown that adult neural crest-derived cells retain the properties of stem cells [16]. Several studies have demonstrated that such neural crest stem cell populations often mimic the transcriptional expression profiles of both embryonic stem cells and neural crest progenitor cells [17]. The presence and wide distribution of neural crest progenitor cells in the tissues of the adult organism are likely to contribute to the appearance of tumors originating from the neural crest.

Multipotent progenitor cells of the neural crest are capable of giving rise to a wide range of tumors in the adult organism. Due to the diverse localization and specification of neural crest progenitor cells, the tumors arising from them constitute a heterogeneous group and can originate in different places throughout the body. These include such widespread adult and pediatric cancers as melanoma and neuroblastoma, as well as other less common types of tumors, namely, paraganglioma, pheochromocytoma, schwannoma, esthesioneuroblastoma, malignant peripheral nerve sheath tumors, granular cell tumors, neurofibroma, perineurioma, neurothekeoma, nerve sheath myxoma, and medullary thyroid cancer [18]. Many researchers suggest that tumors originating from the neural crest may be predisposed to the development of metastatic disease due to the inherent abilities of neural crest cells for self-renewal and migration [19, 20], and indeed, patterns of gene expression and the mechanisms underlying the behavior of the cells of the neural crest and tumor cells have a striking similarity. Expression of different transcription factors critical for neural crest development, including members of the Snail, Twist, SoxE, and FoxD families, is upregulated in many cancers [21, 22]. Both neural crest cells and tumor cells undergo surprisingly similar EMT processes, and both types of cells express matrix metalloproteinases (MMPs) and a disintegrin and metalloproteinases (ADAMs) that facilitate cell invasion and migration [23]. These similarities suggest that the genetic changes underlying the process of tumorigenesis can partially occur due to the reactivation of the factors and signals required for delamination and migration of neural crest cells, which are usually completely suppressed after embryogenesis.

Although the biological aspects of the development and progression of NB have been thoroughly studied for a long time, little is known about the early stages of its pathogenesis, and the cells from which this tumor originates have not yet been reliably identified. Until recently, it was believed that NB originates from the cells of the developing sympathetic nervous system, probably from sympathoadrenal progenitor cells that normally differentiate into sympathetic ganglion cells and adrenal chromaffin cells (catecholamine-secreting adrenal cortex cells) [24]. The sympathoadrenal lineage is derived from neural crest cells that aggregate on the dorsal aorta after migration through the ventral pathway [25]. From the dorsal aorta, these cells then migrate to the developing adrenal glands and become either chromaffin cells or sympathetic ganglia. If the cells differentiate into sympathetic ganglia, they begin to upregulate neuronal markers, whereas chromaffin cells upregulate proteins found in the adrenal glands [26, 27]. Whether a neural crest cell differentiates into a catecholaminergic/adrenal chromaffin cell or a sympathetic neuron depends on the bone morphogenetic proteins (BMPs) [28, 29]. A set of transcription factors, including Sox10 [30, 31] and Mash1 [32], is induced by BMPs, which in its turn regulates the differentiation of migrating neural crest cells into sympathoadrenal cells. The binding of the nerve growth factor (NGF) with the cell-surface receptor TrkA and the permanent presence of the factor are essential for the survival of these differentiated cells, whereas the excess of sympathoadrenal cells undergoes apoptosis on the final stage of differentiation due to the relative deprivation of NGF [33]. A unique feature of neuroblastoma is the mandatory presence of sympathoadrenal neuroblasts, which, unlike normal sympathoadrenal cells, remain viable in conditions of NGF deprivation [34], an experimental fact that confirms the assumption of the origin of neuroblastoma specifically from sympathoadrenal progenitor cells of the neural crest. Moreover, neuroblastoma tumors most often appear in the sites of localization of sympathoadrenal progenitor cells of the neural crest, namely, in the adrenal cortex, the paraspinal ganglia, or the abdominal cavity adjacent to the aorta in the kidney region [35, 36]. There are a number of other facts that directly or indirectly prove that neuroblastoma originates from the progenitor cells of the sympathoadrenal lineage of the neural crest, specifically the following:

- In situ neuroblastomas that arise in the adrenal glands of 1 in 200 newborns and often spontaneously regress afterwards are histologically similar to residual rosette-like sympathogonia arrangements [37].
- Neuroblastomas spontaneously develop in MYCN transgenic mice [38].
- Neuroblastoma cell lines retain the ability to migrate along certain neural crest paths and to colonize tissue targets specific for neural crest cells [39].
- The patterns of gene expression in neuroblastoma are in many respects similar to those of the neural crest progenitor cells [40].

Conversely, Furlan et al. [41] demonstrated the absence of the so-called common sympathoadrenal lineage which gives rise to sympathetic neurons and chromaffin cells in a recent study. The results of this study prove that, in contrast to sympathetic neurons that occur directly from migrating neural crest cells, most of the chromaffin cells (77.8%) in the adrenal medulla are derived from Schwann cell precursors (Figure 1, red arrows). The authors also showed that the separation of these two lineages, sympathetic and adrenal, occurs on the early stages of embryonic development. Also, by use of single-cell RNA sequencing of adrenomedullary cells during the stages of embryonic development, when chromaffin cells only begin to form, the authors have discovered intermediate types of cells that represent successive states between the Schwann cell precursor-to-chromaffin cell transition. Importantly, such intermediate types of cells were not found between Schwann cell precursors and sympathoblast clusters or between chromaffin cells and sympathoblasts. Thus, the authors suggest that neuroblastoma and pheochromocytoma may develop specifically from this chromaffin lineage, since in most cases these tumors are localized in the adrenal gland region.

Members of the MYC family of transcription factors (cMYC, MYCN, and L-MYC) play an important role in cell growth and differentiation [42]. Several studies showed the importance of cMYC on early stages of induction of neural crest progenitors. Knockdown of *cMYC* in chick embryos significantly decreases the number of premigratory neural crest cells, whereas their apoptosis increases considerably. Interestingly, the decrease in the level of cMYC does not affect the proliferation of the neural crest cells but decreases self-renewal of neural crest progenitor cells [43]. The depletion of *cMYC* in Xenopus embryos leads to inhibition of expression or complete absence of early neural crest markers such as slug, twist, and foxd3, as well as to the absence of a different kind of neural crest derivatives characteristic for late stages of embryonic development, namely, mesectodermal neural crest precursors that give rise to the facial skeleton [44]. MYCN expression begins in migrating neural crest cells during the later stages of embryonic development and is accompanied by downregulation of cMYC expression. The main function of MYCN is to preserve the self-renewal and proliferative abilities of neural crest cells [45]. The expression of the *MYCN* oncogene increases during the normal process of sympathoadrenal development and then significantly decreases during the differentiation process [34]. Enhanced expression of MYCN in mature sympathetic neurons induces the progression of the cell cycle and blocks apoptosis [46, 47]. It was shown in E7 chick embryos, when neurogenesis in the sympathetic ganglia reaches its peak, that MYCN expression is limited to SOX2-positive proliferating progenitors, while cMYC is expressed in differentiated Islet1-positive neurons. Overexpression of cMYC and MYCN in chick sympathetic neuroblasts results in a significant increase in their proliferation *in vitro* and *in vivo*, although it does not affect the viability of cells in culture [48]. *MYCN* knockdown in human neural crest stem cells leads to cell cycle arrest in the G0/G1 phase and a significant increase in the expression of *Cdkn1a*, *Cdkn2a*, and *Cdkn2b*, and at the same time a significant decrease in Cyclin D1 expression occurs, which facilitates the G1/S transition [49]. The same members of the MYC family play an important or even a critical role in the development and progression of neuroblastoma. Specifically, the amplification of the *MYCN* oncogene is associated with poor prognosis in patients with neuroblastoma [36]. If not correctly expressed, MYCN can trigger tumorigenesis [38, 50]. Zhu et al. [51] showed that overexpression of MYCN in the zebrafish model terminates chromaffin cell differentiation and facilitates neuroblasts to become hyperplastic. A small part of these neuroblasts eventually forms a heterogeneous tumor [51]. A further study of MYCN, by this time conducted in a murine cell line of neural crest progenitor cells, showed that stable overexpression of either MYCN or ALKF1174L in combination with the absence of cMyc activity leads to the development of a neuroblastoma-like tumor in mice [52]. Primary cultures of mouse neural crest cells overexpressing MYCN also formed neuroblastoma and osteosarcoma in the mouse model. A comparative analysis of human neuroblastoma cells and the neuroblastoma obtained from primary cultures of mouse neural crest cells demonstrated that, in addition to the upregulation of genes associated with the passage through the cell cycle, both neuroblastomas upregulate the transcription factors characteristic of embryonic development of the nervous system, including those important for neural crest development, that is, Sox11, Nhlh2, Twist1, Ascl1, Insm2, and Tcf3 [53].

The anaplastic lymphoma kinase (ALK) gene belongs to the receptor tyrosine kinase (RTK) superfamily. ALK is expressed in the developing central and peripheral nervous system during embryogenesis [54], as well as in the developing sympathoadrenal lineage of the neural crest, where its signalization can regulate the balance between cell proliferation and differentiation [55–57]. The physiological role of ALK in the course of normal development of the nervous system is not yet fully understood; however, the importance of ALK in neurogenesis of *Drosophila*, zebrafish, and chicken has been proved [56, 58, 59].

It has been recently shown that ALK expression is associated with a less differentiated state of neuroblastic tumors, and the incidence of ALK overexpression in neuroblastoma is significantly higher than in more differentiated ganglioneuroblastoma and ganglioneuroma [60, 61]. Due to the fact that frequent ALK-wt overexpression in primary neuroblastoma tumors was associated with a poor clinical outcome, similarly to the presence of activating ALK mutations such as *ALK-F1174*L and *ALK-R1275Q* [62–64], it was suggested that excess expression of ALK-wt may be involved in oncogenesis and progression of neuroblastoma. Montavon et al. [65] demonstrated that expression of ALK-wt in JoMa1 and MONC-1 cell lines may lead to the formation of malignant tumors in neural crest progenitor cells of nude mice in the same way as the expression of activating *ALK-R1275Q* and *ALK-F1174*L mutations. These results prove that a few genetic modifications are enough for progenitor cells of the neural crest to become malignant.

**Figure 2:**
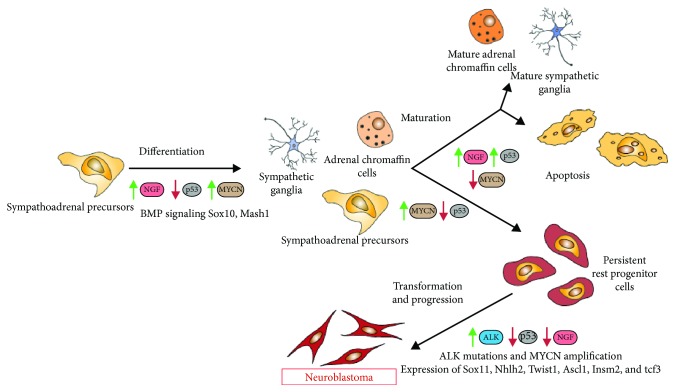
The transformation of neural crest cells into neuroblastoma cells.

Sox2 and Nanog are the most important factors necessary for maintaining the ability of stem cells to self-renew. Pandian et al. [66] demonstrated that the expression levels of both of these proteins are also significantly increased in cancer stem cells of neuroblastoma. Quantitative transcriptional profiling showed an increase in the expression of 29 molecules, including BMPs, Notch2, Slug, and Twist1, associated with the stem state in cancer stem cells of neuroblastoma. The authors also described high levels of cellular plasticity and increased pluripotency of aggressive metastatic neuroblastoma cells as compared to the original cell lines. Together, these data indicate that many of the proteins that play an important role in maintaining the potency of embryonic stem cells, including neural crest cells, can perform similar functions in controlling and maintaining the potency of cancer stem cells. The main factors that determine the transformation of neural crest cells into neuroblastoma cells are shown in Figure 2.

Thus, the abovementioned data demonstrate that (i) neuroblastoma originates from progenitor cells of the neural crest and (ii) there is a significant molecular similarity between the cells of the most aggressive undifferentiated neuroblastoma and early embryonic progenitor cells.

## 3. Tumor Markers in Neuroblastoma

Neuroblastoma is a highly heterogeneous tumor. A single tumor may contain a wide range of cellular phenotypes characteristic of this transient embryonic structure, in particular neuroblasts, melanocytes, and nonneuronal cells such as Schwann, perineurial, or satellite cells. It was shown that the heterogeneity of neuroblastoma and the degree of maturity of the cells that form it (e.g., stroma-poor tumors as opposed to stroma-rich tumors, or high-risk versus low-risk tumors based on histological gradation) correlate with clinical behavior and prognosis. These characteristics are used for both the classification of the tumor and the prognosis of the disease [67]. On the other hand, van Groningen et al. [68] have shown that neuroblastoma tumors are not as heterogeneous as was previously thought. The authors found that most neuroblastomas include two major types of tumor cells with divergent gene expression profiles, namely, undifferentiated “mesenchymal” cells and committed adrenergic cells that can transform into each other but resemble normal cells at different stages of differentiation. Cells of the “mesenchymal” type showed higher resistance *in vitro* to standard neuroblastoma chemotherapy drugs, such as cisplatin, doxorubicin, and etoposide, as compared to isogenic adrenergic cells. Moreover, biopsy material obtained from the patients with neuroblastoma after chemotherapy or after relapse was significantly enriched with “mesenchymal” tumor cells [68].

Boeva et al. [69] have demonstrated a new type of heterogeneity in neuroblastoma cell lines and primary tumors by identifying three types of cells: sympathetic noradrenergic cells that are characterized by expression of PHOX2B, HAND2, and GATA3 transcription factors; neural crest cell-like (NCC-like) cells that express FOS and JUN family members but do not express PHOX2B and noradrenergic markers; and a mixed type of cells. All cell lines with MYCN amplification except CHP-212 belonged to the noradrenergic type, whereas all three types were represented among the cell lines without MYCN amplification. Similar results were obtained for primary tumor samples. Enrichment of the cell population with NCC-like cells correlated with a stronger resistance to chemotherapy. Still, the authors do not rule out the possibility of transdifferentiation of noradrenergic cells into NCC-like cells during chemotherapy.

Certain heterogeneity is also observed in the cell lines obtained from neuroblastoma tumors. Three different phenotypes were identified in most studies conducted on established human neuroblastoma cell lines [70, 71]. Sympathoadrenal neuroblasts (N-type cells) that have small rounded cell bodies with short neurites and express neuronal cell markers are the most common type; these cells are either attached to the substrate in a small degree or grow in free-floating clusters in culture. N-type cells are weakly tumorigenic: they form slowly growing tumors in 30% to 100% of mice and show a minor ability to grow in soft agar. The second type of cells is represented by nonneuronal precursor cells that are strictly substrate-attached, grow as a contact-inhibited monolayer, and have a high cytoplasm/nucleus ratio. These cells are called S-type cells (substrate-adherent); they are large in size and have a flattened morphology. S-type cells express marker proteins that identify them as nonneuronal cells originating from the neural crest, such as melanocytes and glial and smooth muscle cells. Cells with this phenotype are nontumorigenic: they do not exhibit at all or exhibit an insignificant degree of anchorage-independent growth in soft agar and do not form tumors in nude mice [72]. The third type of cells called I-type cells due to an intermediate morphology between N- and S-type cells are actually stem-like cells. I-type cells are small, flattened, moderately adherent cells with or without neurites that form aggregates in culture. These highly primitive stem cells that are capable of self-renewal and bidirectional differentiation can develop into either N- or S-type cells [73]. I-type cell lines express marker proteins of both N and S phenotypes. Of particular interest is the fact that I-type cells are 4-5 times more tumorigenic than N-type cells are [74]. Moreover, the higher malignant potential of I-cells does not depend on the status of amplification of *MYCN*, which is usually an indicator of aggressive behavior of the tumor [71]. Ross et al. [75] found 7 genes which were more than five-fold overexpressed in I-type cells compared to N- or S-type cells: CD133, KIT, NOTCH1, GPRC5C, PlGF2, LNGFR, and TRKB.

Due to the fact that malignant stem-like cells were found in many neuroblastoma cell lines, it was suggested that a similar pool of cells with increased malignancy and aggressiveness is present in neuroblastoma tumors. Hirschmann-Jax et al. [76] characterized primary tumor cells obtained from 23 patients on different stages of neuroblastoma (17 patients with stage IV, 5 patients with stage III, and 1 patient with stage I) for the purpose of identificating a side population (stem-like cell population). Side population cells were found in 15 of 23 (65%) neuroblastoma samples. The phenotypic analysis of surface markers expressed by side population cells showed an increased number of cells expressing ganglioside GD2 and c-kit (stem cell factor receptor, CD117) with a higher intensity than the cells not included in the side population. Cells of the side population also expressed low levels of CD133, CD71, CD56, and high levels of transporter proteins ABCG2 and ABCA3, which alongside others are responsible for the resistance of cancer cells to antineoplastic drugs such as mitoxantrone [77, 78].

Walton et al. demonstrated the presence of I-type cells in tumor samples obtained from patients with relapsed neuroblastoma and patients without disease progression. The authors performed immunohistochemical staining of cryosections from neuroblastoma tissues using antibodies specific for markers of S- and N-type cells, S100A6 (calcyclin) and neurofilaments, respectively. Cells expressing both markers simultaneously were considered I-type cells. It was found that the putative I-type cancer stem-like cells are present in all analyzed samples at a frequency of 1% to 90%. Besides, the authors observed a strict correlation between relapse and the incidence of I-cells in the samples. The frequency of I-cells in tumors from patients with relapse disease was almost five times as high as in patients without disease progression [71].

As has been shown in multiple studies, despite the tumor's heterogeneity, there is a pool of cells in neuroblastoma, the so-called cancer stem cells or more correctly tumor-initiating cells [79], which display increased aggressiveness and tumorigenicity and promote tumor development and metastasis. Their presence in the tumor foci and/or in metastases correlates with the stage of the disease, the risk of development (low-risk or high-risk neuroblastoma), and poor clinical outcome. That is why searching for markers specific for this pool of cells that could be used for targeted high-performance therapy of high-risk neuroblastoma becomes an urgent and important task.

### 3.1. C-Kit/SCF

The *c-kit* gene encodes the transmembrane receptor CD117 that contains a tyrosine kinase component. The stem cell factor (SCF) binds to c-kit resulting in the signaling via the SCF/c-kit pathway, which is important in hematopoiesis, gametogenesis, and melanogenesis [80]. The so-called gain-of-function somatic mutations that lead to constitutive activation of c-kit are observed in several malignant diseases, such as gastrointestinal stromal tumors [81], mastocytosis [82], acute myeloid leukemia [83], and testicular seminoma [84], whereas in the cases of small cell lung cancer [85], neuroblastoma [86], colorectal cancer [87], and ovarian cancers [88], mutations in *c-kit* have not been identified, but paracrine and/or autocrine activation of c-kit does occur during transformation and progression.

The function of c-kit and SCF in the development and progression of neuroblastoma has not been reliably established to date. The available literature provides very contradictory data on the prognostic role of SCF/c-kit regarding clinical characteristics of the tumor and results of neuroblastoma therapy. Several studies demonstrated that there was no correlation between SCF/c-kit expression and clinical characteristics [86, 89]. Thus, Cohen et al. [86] analyzed the joint expression of SCF and c-kit in 14 neuroblastoma cell lines and 18 samples of tumor material. All cell lines examined in this study expressed, to varying degrees, both SCF and c-kit, and the level of c-kit expression in the original cell lines and in the N-clones obtained from them was significantly higher than in the I- or S-clones. The antibodies that block c-kit significantly reduced the proliferation and clonogenic potential of neuroblastoma cell lines. Only 8 of 18 (45%) tumor samples obtained from patients with neuroblastoma expressed both SCF and c-kit, and none of the samples expressed only one of the proteins. No significant correlation between SCF/c-kit expression and stage of disease, *MYCN* amplification, and other clinical indices was determined, which is probably due to a small sample of patients. In another study, Beck et al. [89] showed low constitutive expression of SCF in 7 of 8 neuroblastoma cell lines, and only one of them expressed c-kit. Analysis of tumor samples showed a low frequency of anti-c-kit antibody immunoreactivity in primary neuroblastoma tumors or ganglioneuromas and a variable immunoreactivity in metastatic samples; besides, no coexpression of c-kit and SCF was observed. Only a few neuroblastoma samples showed weak and diffuse expression of SCF, whereas three ganglioneuroma samples displayed focal and intensive SCF expression. Based on the results, the authors deny the presence of an autocrine SCF/c-kit loop in neuroblastoma.

More recent studies showed a correlation between the expression of SCF/c-kit and the clinical characteristics of neuroblastoma tumors; however, contradictions regarding whether the correlation is positive or negative still exist. Particularly, Krams et al. [90] found a correlation between the expression of c-kit in neuroblastoma samples and a favorable prognosis for patients. Expression of this protein correlated with a lower stage of the disease and a low level of *MYCN* amplification, whereas most of the c-kit-positive neuroblastomas represented differentiated (44.7%) or slightly differentiated (18.8%) tumors.

In contrast to this, in a study by Uccini et al. [91], expression of both c-kit and SCF correlated with unfavorable clinico-biological characteristics. C-kit protein expression was detected in 21 of 168 (13%) neuroblastoma samples; mRNA expression in 23 of 106 (22%) samples. The SCF protein was detected in 30 of 106 (28%) samples; its mRNA in 30 of 106 (31%) neuroblastoma samples. As a rule, c-kit- and/or SCF-positive cases were undifferentiated or slightly differentiated neuroblastomas at a late stage (III and IV) and with unfavorable molecular characteristics, such as *MYCN* amplification and 1p36 allelic loss. In addition, the survival rate of patients with c-kit-positive tumors was 17%, while the survival rate of patients with c-kit-negative tumors was 68%. For SCF, these values were 43% and 78%, respectively.

In another study conducted on a wide range of neuroblastoma cell lines, overexpression of the *c-kit* gene was most often associated with the amplification of the *MYCN* gene [92]. Imatinib mesylate, a selective inhibitor of c-kit and PDGFR protein kinases, suppresses the proliferation of neuroblastoma cell lines *in vitro* [93, 94] and inhibits the tumor growth of c-kit/PDGFR-positive neuroblastoma xenografts *in vivo* [95]. It has also been shown that downregulation of c-kit in the neuroblastoma cell line SH-SY5Y stimulates the expression of genes that can be involved in spontaneous tumor regression or in its differentiation. Specifically, downregulation of c-kit increased the expression of the neurotrophin TrkA receptor, which is a prerequisite for spontaneous regression of neuroblastoma, and also increased expression of HLA class I genes and levels of IFNGR expression, which significantly raises the sensitivity of tumor cells towards the components of the immune system [96].

There are currently no immunotherapeutic approaches in the clinic that use c-kit as a target for the therapy of any disease, including cancers. The most widely investigated area in the context of c-kit-positive tumors is the use of small molecule inhibitors which are selectively targeted at suppressing the signaling through this tyrosine kinase receptor. Tyrosine kinase inhibitors demonstrate significant clinical success, especially in the treatment of gastrointestinal stromal tumors, where imatinib is currently the standard therapy for patients with high-risk disease [97]. Despite their effectiveness, the use of these inhibitors invariably leads to the development of resistance, often due to mutations occurring in the kinase domain, which interfere with the binding of the drug [98]. In addition, some tumors including neuroblastoma exhibit aberrant c-kit expression in the absence of gene mutations, which significantly reduces the activity and effectiveness of the inhibitors. Recently, several studies appeared in the literature demonstrating the antitumor activity of c-kit-specific antibodies that block the interaction of the receptor with its ligand SCF. One of these antibodies, ACK2, showed antitumor activity in RET transgenic mice, in which melanoma develops spontaneously after birth. A single injection of anti-c-kit antibodies shortly after birth resulted in a prolonged suppression of melanoma, a greatly increased tumor-free period, and none of the animals injected with anti-c-kit antibodies died from cancer for 12 months after birth [99]. Another group of researchers developed humanized anti-c-kit monoclonal antibodies, KTN0158, that specifically interact with both mutant and wild-type c-kit and exhibit biological activity in normal and malignant mast cells [100]. In 2015, Kolltan Pharmaceuticals launched a phase I clinical trial of anti-c-kit monoclonal antibodies, KTN0158, as a monotherapeutic agent for the treatment of patients with gastrointestinal stromal tumors and other c-kit-positive tumors (NCT02642016). In addition, the company is developing approaches to use these antibodies in combination with immune checkpoint inhibitors (anti-CTLA4 and/or anti-PD1/PD-L1 antibodies), which are based on the potential of anti-c-kit antibodies to modulate the relationship of the immune system and the tumor [101]. The inhibition of c-kit using specific antibodies resulted in a noticeable decrease in the amount of myeloid-derived suppressor cells (MDSC) in the tumor microenvironment and significantly increased the antitumor activity of T cell checkpoint antibody-based inhibitors [102].

Thus, despite a number of contradictions regarding the correlation of c-kit expression with the clinico-biological characteristics in such a complex tumor as neuroblastoma, the tyrosine kinase receptor c-kit can potentially serve as a target for antitumor therapy of this cancer, at least as part of a combination therapy to enhance efficacy of other approaches.

### 3.2. CD133

CD133 (prominin-1) is a transmembrane protein with a molecular weight of 120 kD, which was initially viewed as a hematopoietic stem cell marker [103]. It was also shown that CD133 is expressed by various human cell lines [104], colorectal cancer cells [105], neural stem cells [106], and other cancer stem cells from different types of tumors [107, 108]. Some studies propose CD133 as an independent prognostic marker of low survival in colorectal cancer [105], hepatocellular carcinoma [109], and glioma [110]. CD133 is also a marker of human neural crest stem cells [111], so it can be assumed that CD133-positive stem cells may serve as an indicator of tumors originating from neural crest cells. Much reliable data has emerged lately showing that CD133 not only is a specific biomarker of different types of stem cells but also plays a key role in the processes of cell growth, development, and tumorigenesis [107].

It has been shown in different studies that expression of CD133 is a sign of an unfavorable outcome in a number of cancers, including neuroblastoma [112]. CD133 overexpression is associated with increased chemoresistance [68, 113] and inhibits differentiation of neuroblastoma cells [114]. For neuroblastoma cell lines, it was demonstrated that CD133 expression is approx. five times higher in I-type cells, which are regarded as cancer stem cells, compared to N- and S-type cells [75]. Several independent studies examined the relationship between CD133 expression and the stage of neuroblastoma [68, 112, 115]. Mehrazma et al. [115] found no expression of CD133 in the majority of patients with stage I neuroblastoma and in 1 of 8 patients with stage IV neuroblastoma, whereas in 7 of 8 patients with stage IV neuroblastoma the intensity of CD133 expression was high. In this case, the authors suggested that there is a significant relationship between the stage of cancer and the level of CD133 expression and that the CD133 expression is higher in late-stage cancer. Similar results were obtained in another study where expression of CD133 was shown in 84 of 106 patients with type IV neuroblastoma and in 37 of 97 patients with neuroblastoma metastases [68]. A positive correlation between CD133 expression and the stage of cancer was also shown by Tong et al. [112] in an earlier study. Both of the abovementioned investigations [68, 112] reliably demonstrated that CD133 expression is significantly higher in patients with stage III–IV neuroblastoma, and the increase in the expression of this marker correlates with tumor progression. Moreover, Sartelet et al. [113] established that CD133 expression is associated with shorter patient survival.

van Groningen et al. [68] generated new neuroblastoma cell lines from the biopsy material of patients with neuroblastoma. Despite their genetic identity, the cell lines obtained from the same patient showed divergent phenotypes and mRNA profiles. The *PROM1* gene that encodes CD133 was one of the differentially expressed genes. CD133^+^ cells grew as an adherent monolayer, formed lamellipodia, and displayed mobility, whereas CD133^−^ cells formed semiadherent spheres and were not capable of migration. CD133^−^ cells expressed genes involved in adrenergic differentiation, such as *PHOX2A*, *PHOX2B*, and *DBH*. The authors termed these cells as adrenergic neuroblastoma cells. In contrast, CD133^+^ cells expressed mesenchymal markers SNAI2, vimentin, and fibronectin and were termed as “mesenchymal” tumor cells. Both types of cells are capable of transdifferentiating into each other. Although no significant correlation between the numbers of CD133^+^ “mesenchymal” cells and clinical and molecular characteristics of neuroblastoma was shown, these cells displayed increased resistance to chemotherapy and were significantly more prevalent in tumor samples after chemotherapy in the cases of relapse cancers compared to CD133^−^ adrenergic cells [68]. The progressive increase in the number of CD133^+^ cells has also been shown *in vitro* in neuroblastoma cell lines after several cycles of chemotherapy treatment [116]. Zhong et al. [117] established a correlation between CD133 expression, *MYCN* amplification, chemoresistance, and the survival of neuroblastoma patients. Only 8% of CD133^+^ neuroblastoma patients showed complete response to chemotherapy, whereas CD133^−^ patients demonstrated a complete response in 21% of cases. The expression of CD133 did not significantly affect the overall patient survival; however, the survival rate of CD133^+^ patients with amplified *MYCN* was considerably low (12.5 months) [117].

Despite the fact that CD133 is a proven and functionally active marker of cancer stem cells, including neuroblastoma, clinical approaches using this molecule as a target for anticancer therapy have not yet been developed. Nevertheless, it is proved that CD133 can serve as a direct target for biological drugs on the surface of cancer stem cells, leading to the effective and selective elimination of these cells. For this reason, the first attempts have recently been made to develop anti-CD133 therapy for oncological diseases where cancer stem cells overexpress this marker. Vallera and colleagues showed that the fusion protein CD133KDEL, consisting of a CD133-specific scFv fragment and a deimmunized form of pseudomonas exotoxin A, has a high antitumor and cytotoxic activity in various xenograft cancer models, including head and neck cell carcinoma [118], breast carcinoma [119], and ovarian cancer [120]. Polymer nanoparticles loaded with paclitaxel and targeted to CD133 (CD133NPs) on Caco-2 colorectal adenocarcinoma cells were shown to effectively reduce the number of cells and the formation of colonies *in vitro* and also demonstrated better therapeutic effects in the xenograft model of breast cancer compared to the treatment with free paclitaxel [121]. Also, anti-CD133 immunotoxin therapy has been tested on cancer stem cells of sarcoma [122]. These studies suggest that anti-CD133 therapy can be based on the delivery of drugs directly to cancer stem cells, which leads to their more effective elimination. A different approach that could employ the CD133 marker in anticancer therapy is the development of bispecific antibodies, in which one activity is directed to the CD133 antigen, and the other towards the activation of immune cells. Huang et al. [123] showed effective killing of pancreatic and liver cancer cells that express high CD133 levels *in vitro* as well as inhibition of tumor growth *in vivo* by cytokine-induced killer cells associated with bispecific anti-CD3/anti-CD133 antibodies. Schmohl et al. [124] developed heterodimeric bispecific single-chain variable fragment killer engagers (BiKEs) that simultaneously recognize the tumor-specific CD133 and CD16 on NK cells, and showed a significant increase in NK-mediated killing of human Caco-2 colorectal adenocarcinoma cells that overexpress CD133, as well as the enhancement of NK cell cytotoxicity against the NK-resistant human Burkitt's lymphoma Daudi cell line, in which less than 5% of cells express surface CD133. An alternative approach for anti-CD133 therapy is the modification of immune cells with chimeric antigen receptors, specifically the generation of CAR T cells. It was shown that CD8^+^ CAR T cells specific to the AC133 epitope of CD133 effectively eliminate CD133^+^ stem cells from glioblastoma multiforme *in vitro* and in the orthotopic tumor model *in vivo* [125].

Based on the experimental studies using the surface molecule CD133 as a target for anticancer therapy, it can be concluded that this area of research is quite promising in view of encouraging results obtained from animal tumor models. These results may lead to the creation of approaches specifically aimed at elimination of cancer stem cells that are responsible for tumor maintenance, development, and metastasis.

### 3.3. CD114

CD114 is one of the cancer stem cell markers originating from the neural crest, which acts as the transmembrane receptor for the granulocyte colony-stimulating factor (G-CSF). CD114^+^ neuroblastoma cells are highly tumorigenic and are characterized by chemoresistance and the ability to self-renew and differentiate into all types of cells that make up the tumor. Gene expression profiles in CD114^+^ cells are similar to those of embryonic and induced pluripotent stem cells. These cells are found in neuroblastoma cell lines, in murine xenograft tumors, and in primary tumor samples taken from patients with neuroblastoma [126–128]. However, despite the expression of CD114 in tumors of various origins, its role and function in the development of malignancies have not been established yet.

The protumorigenic role of G-CSF was demonstrated in many studies [129, 130]. Due to the fact that CD114 is the marker of both neural crest progenitor cells and cancer stem cells originating from the neural crest, it is an excellent target for the development of new therapeutic approaches. Currently, large quantities of G-CSF are being used to treat neuroblastoma patients to ensure bone marrow recovery after several cycles of myelosuppressive chemotherapy, although sufficient evidence has not yet been obtained to confirm that such therapy improves overall patient survival [131]. Since it has been proved that G-CSF stimulates proliferation and tumor growth and increases the chemoresistance of neuroblastoma cancer stem cells, the routine use of this growth factor for the treatment of patients with neuroblastoma may lead to undesirable consequences, aggravating the patients' condition. Therefore, it is essential to search for alternative approaches aimed at CD114/G-CSF [18].

### 3.4. CD57

CD57 (Leu-7/HNK-1) is a carbohydrate epitope expressed on adhesion molecules of migrating neural crest progenitor cells [132, 133]. It has been shown that CD57 mediates invasion and migration of neural crest cells [132, 133] and is associated with metastasis in melanoma, a tumor that, like neuroblastoma, originates from neural crest progenitor cells [134]. This marker is used alongside with CD56 in the diagnostics of cancer by immunostaining methods to differentiate neuroendocrine tumors from other types of tumors. A flow cytometry assay showed that 10 out of 15 bone marrow metastasis samples from neuroblastoma patients were positive for CD57 [135]. Also, in another work, CD57 was immunohistochemically stained in 100% of poorly differentiated neuroblastoma, 96% of differentiated neuroblastoma/ganglioneuroblastoma, and 93% of ganglioneuroma. Moreover, in the same study 100% of bone marrow metastases were stained with CD57-specific antibodies [136]. Schlitter et al. [137] showed that, regardless of the amplification of *MYCN*, CD57^high^ neuroblastoma cells exhibit more aggressive features compared to CD57^low^ cells. CD57^high^ neuroblastoma cells demonstrated increased ability to form spheres and invade the extracellular matrix, as well as to adhere to endothelial cells and penetrate the basal membrane *in vitro*. These properties define CD57^high^ neuroblastoma cells as more aggressive compared to CD57^low^ cells. In addition, CD57^high^ cells were phenotypically immature and were more likely to form metastases in the liver after intravenous administration to experimental animals in comparison with CD57^low^ cells. The authors also showed that high levels of CD57 expression in samples of primary neuroblastoma are strictly associated with undifferentiated neuroblastoma cells, and the frequency of CD57^high^ cells increases after chemotherapy [137].

### 3.5. CD171

Aberrant regulation of adhesion molecules that contributes to the progression of the oncologic process often occurs during malignant transformation of cells. L1CAM (L1 cell adhesion molecule) or CD171 is one of these molecules. L1CAM is a protein with a molecular weight of 200–220 kDa, which is a member of the immunoglobulin superfamily of cell adhesion molecules (IgCAMs). Initially, the L1CAM molecule was identified in the nervous system [138]. The extracellular part of L1 consists of 6 Ig-like domains and 5 fibronectin-type III domains and is connected to a short intracellular cytoplasmic domain through a single transmembrane sequence [139]. The functioning of L1CAM largely depends on the homophilic and heterophilic interactions of its extracellular domains with other cell adhesion molecules on the cell surface [140].

Normally, L1CAM plays a key role in the development of the nervous system, regulation of intercellular interactions, migration of nerve cells, growth of neurites on Schwann cells, myelination of nerve fibers, and so on [141–143]. Aberrant expression of L1CAM is found in many types of human cancers, including colorectal cancer [144], melanoma [145], breast cancer [146], ovarian carcinoma [147], kidney carcinoma, neuroblastoma [148], gastrointestinal stromal tumors (GISTs) [149], pancreatic carcinoma [150], Schwannoma [151], and glioma [152]. A multiple tumor tissue microarray conducted on 128 different types of tumors showed that most of L1CAM-positive tumors have neuroectodermal and neural crest origin, and among these tumors, 96% neuroblastomas, 93% granular cell tumors, 76% pheochromocytomas, 86% schwannomas, 54% primitive neuroectodermal tumors, 40% paragangliomas, and 56% of GISTs are strictly positive for L1CAM [153]. Expression of L1CAM in cancer tissues and cultured tumor cells in most cases correlates with poor clinical prognosis and a late stage of the disease. In normal human tissues, L1CAM is detected in small amounts in basal cells of the skin, endothelial cells of small blood vessels, mature placenta, renal ducts, and peripheral nerves. These facts make L1CAM a promising marker in the diagnostics and therapy of L1CAM-expressing tumors [154].

The L1 cell adhesion molecule was discovered on neuroblastoma cells in the 1980s [155, 156]. Two isoforms of L1 are expressed on neuroblastoma cells—the full-length L1CAM molecule and the L1CAM variant without exons 2 and 27. The role of L1CAM in tumor cells is to maintain tumor growth, metastasis, and angiogenesis. It has been shown in the neuroblastoma cell line IMR-32 that the knockdown of L1CAM leads to a decrease in the formation of tumorospheres, a decrease in the proliferation and migration of tumor cells, and downregulation of MYCN and upregulation of the PTEN tumor suppressor. L1 knockdown also increased the radiosensitivity of tumor cells [157].

Despite the fact that L1CAM expression is a poor prognostic factor for various types of tumors, the situation is not so obvious for neuroblastoma. So, for example, Wachowiak et al. [158] analyzed L1CAM expression using tissue microarray with 66 surgically resected neuroblastoma samples and showed that L1 expression is a factor of positive prognosis for patients with neuroblastoma. Still, most authors who conducted large-scale studies of L1CAM expression in tumors of various origin, including neuroblastoma, concur that expression of this marker is rather a prognostic factor of poor outcome [159].

Regardless of these contradictions, active development of various therapeutic approaches using the L1CAM molecule as a diagnostic and therapeutic marker for the treatment of neuroblastoma is underway. L1CAM serves as a target for the treatment of various types of cancer using monoclonal antibodies and antibody fragments, and neuroblastoma is not an exception in this context. For example, chimeric antibodies chCE7 targeting L1CAM that were developed in the 1980s [160] bind the CE7 epitope of L1 and are internalized by neuroblastoma and renal carcinoma cells [161]. сhCE7 also inhibits the proliferation of L1CAM-positive tumor cells, namely, neuroblastoma, kidney carcinoma, colon cancer, and ovarian cancer. In addition, 131 iodine-labeled chCE7 antibodies were successfully used to visualize the tumor in a xenograft model [162] and in patients with recurrent neuroblastoma [163] and also showed an antitumor effect in a xenograft model, inducing an almost complete elimination of subcutaneous tumor lesions.

A number of studies demonstrated that the effect of different anti-L1CAM antibodies on tumor cells can be utterly varying (from inhibition to stimulation of tumor cells), which is mainly due to the particular epitope that the antibody binds. Wang et al. [164] developed scFv fragments specific to different L1 domains and showed in a neuroblastoma cell line that some antibody fragments have a stimulating effect, and others have an inhibitory effect on tumor growth. So, the scFv fragments I4 and I6, specific to the immunoglobulin domains 1–4 of the L1 molecule, inhibited the proliferation of SK-N-SH neuroblastoma cells, while the scFv-fragments I13 and I27, specific for the fibronectin type III (Fn3) homologous domains 1–3, enhanced the proliferation of these cells *in vitro*.

scFv fragments of chCE7 antibodies are widely used in the development of CAR T cells targeted to solid tumors, including neuroblastoma [165]. The chimeric immunoreceptor-designated CE7R specifically targets the CE7 epitope of L1 on neuroblastoma cells and also the cells of the adrenal medulla and sympathetic ganglia which show a limited expression of the antigen.

CE7R-modified cytotoxic T cells are redirected to L1CAM-positive human neuroblastoma cells and activated for tumor cell lysis and TC-1 cytokine production [166]. Park et al. [167] conducted a phase I clinical trial using autologous CE7R/HyTK^+^ CD8^+^ cytolytic T lymphocytes to treat patients with advanced recurrent metastatic neuroblastoma. CAR T cells used in the trial were safe but displayed limited effectiveness. Patients who were included in the trial but did not receive T cell therapy died within 337 days after the beginning of the trial. Only one out of six patients receiving CARTs showed disease stabilization and then a partial response after the first infusion of T cells. Following additional therapy, one patient had a complete response and another patient showed stabilization of the disease. Only one patient out of six receiving adoptive T cell therapy showed survival prolongation with relapse after 4.5 years. Low antitumor efficacy of T cell therapy is probably associated with the absence of CD4^+^ helper cells and costimulation, since the trial employed first-generation CARs. In order to improve therapeutic efficacy, CE7-CARs containing a short extracellular domain [168] and one (4-1BB; second generation (2G)) or two (CD28 and 4-1BB; 3G) intracellular costimulatory signaling domains were developed. All variants of CE7-CAR T cells showed antitumor effects *in vitro* and *in vivo* in preclinical studies, and a phase I clinical trial of 2G and 3G CE7-CAR T cells was initiated in patients with refractory or relapsed neuroblastoma (NCT02311621; INDFDA number 16139) [169].

In conclusion, due to the uniform and abundant expression of CD171 on the surface of the neuroblastoma cells, this antigen appears to be a very promising target for immunotherapy, especially for the application of CAR T cells.

### 3.6. Tumor-Associated Gangliosides

At present, tumor-associated gangliosides are possibly the most promising, widely studied, and already actively used neuroblastoma markers in the clinic. Gangliosides are universal components of eukaryotic cell membranes. They belong to the family of glycosphingolipids (GSL) and contain one or more sialic acids, N-acetyl derivatives of neuraminic acid, in their hydrophilic oligosaccharide chain. The ceramide, which serves as the hydrophobic part of the ganglioside, consists of a long-chain sphingosine or sphinganine linked to a fatty acid by an amine bond. The ganglioside uses the ceramide to anchor in the outer leaflet of the plasma membrane of cells and displays its hydrophilic part to the extracellular space. Such positioning of gangliosides in the plasma membrane determines their basic biological functions. Gangliosides participate in cellular communication, adhesion, growth, and differentiation [170, 171]; play an important role in cellular recognition [172]; modulate signal pathways through glycosynapses [173]; mediate contact inhibition; and participate in the formation of lipid rafts that regulate the activity of various cellular processes [174, 175]. Gangliosides can also take part in inhibiting the proliferation of T and NK cells [176–178]. The composition and metabolism of GSL constantly change depending on the processes taking place in the cell. These specific changes occur during cell proliferation [179] and differentiation, during cell passage through different phases of the cell cycle [180], and during embryonic development of the brain [181] and malignant transformation [182]. The most significant changes in the metabolism of gangliosides and, as a consequence, in the patterns of their expression occur during malignant transformation of cells. Due to this fact, a number of gangliosides are considered tumor markers of various geneses.

Malignant transformation of cells is accompanied by an aberrant composition of the cell surface, which is closely related to the abnormalities in the glycosylation pathways of glycoconjugates and, in particular, gangliosides [183]. Since gangliosides are the main components of the cell surface glycocalyx and are involved in intercellular interactions and cell-matrix interactions, they are also involved in the invasive/metastatic behavior of tumor cells [184, 185]. Changes in the composition of gangliosides and their structure in the process of neoplastic transformation were demonstrated more than 30 years ago [186–188]. Comparison of tumor tissues with respective normal tissues showed the change in the overall level of ganglioside expression as well as emergence of new types of GSL that are absent or rarely expressed on normal cells. In general, increased expression of simple gangliosides, including GM3, GM2, GD3, and GD2, characterizes aggressive tumors such as melanoma and neuroblastoma [187, 189–191]. The correlation between ganglioside expression and the age of neuroblastoma patients was demonstrated; higher levels of the “b-series” gangliosides (GD3, GD1b, GT1b, and GQ1b) are mainly characteristic for newborn patients with neuroblastoma as compared to older patients [192]. Some tumor-associated gangliosides may serve as prognostic factors in neuroblastoma. Decrease or absence of expression of “b-series” gangliosides, GD1b, GQ1b, and GT1b, correlates with reduced survival of patients with neuroblastoma [190, 193] and with a more aggressive biological tumor phenotype [194]. Overexpression of the complex gangliosides GM1, GD1a, GD1b, and GT1b in neuroblastoma cells significantly inhibits their ability to migrate and, therefore, prevents tumor metastasis [195]. And indeed, it was shown that high expression of complex b-series gangliosides strongly correlates with a favorable outcome [194, 196]. Thus, it has been repeatedly proved that ganglioside metabolism can vary depending on the degree of malignancy of neuroblastoma and can affect the clinical picture and outcome for patients [190, 197]. These facts make gangliosides good prognostic markers predicting the clinical outcome for neuroblastoma patients.

Shedding of tumor-associated gangliosides from the cell surface was demonstrated for a large number of tumor cells, including those of neuroblastoma [198], lymphoma [199], melanoma [200], leukemia [201], and brain tumors [202]. Tumor gangliosides freely circulating in the blood inhibit certain immune responses both *in vivo* and *in vitro* [203, 204]. They may specifically cause local immunosuppressive effects [205], inhibiting the proliferation of T lymphocytes [206, 207] as well as IL-2-dependent responses [208]. Their shedding from the surface of transformed cells stimulates the development of tumors in mice [209] and humans [210].

Neuroblastoma tissues overexpress the simple b-series ganglioside GD2 [187]. Normally, GD2 is expressed in the cells of the central nervous system [211], peripheral neurons [212], melanocytes, and bone marrow mesenchymal stromal cells [213]. In neuroblastoma tumors, GD2 is expressed in neuroblastic cells, but not in Schwannian stromal cells, at a high level [214] and along with ganglioside GD3 is involved in adhesion of tumor cells to the extracellular matrix [215]. GD2 is a good diagnostic marker: it makes possible to differentiate neuroblastoma from benign ganglioneuroma and intermediate-grade ganglioneuroblastoma tumors that do not express or express this ganglioside at a low level. Expression of GD2 is an indicator of neuroblastoma, and high levels of GD2 shedding into the circulation correlate with faster disease progression [210]. However, the use of the ganglioside GD2 as a prognostic marker may not be appropriate in all cases, since no correlation has yet been found between the expression of GD2 and such important clinico-biological parameters as the age of the diagnosis, the clinical stage, and the amplification of *MYCN*. At the same time, in patients with neuroblastoma, shedding of tumor gangliosides directly correlates with the degree and rate of tumor development; patients with high circulating GD2 levels (>568 pmol/ml) have a shorter average progression-free survival (about 9 months) compared to patients with low GD2 levels (<103 pmol/ml, 28 months) [210]. Still, evidence exists that the level of expression of GD2 on neuroblastoma tumor cells may serve as a prognostic factor for immunotherapy with GD2-specific antibodies. Terzic et al. [214] demonstrated that expression of GD2 in primary tumors of high-risk neuroblastoma patients without relapse was significantly higher than in primary tumors of patients with disease relapse after treatment with anti-GD2 monoclonal antibodies. These results suggest that resistance to anti-GD2 immunotherapy may be due to the presence of GD2-negative cells in primary tumors or a low expression level of this ganglioside.

Ganglioside GD2 is the primary molecular target for neuroblastoma immunotherapy. The GD2-specific monoclonal antibody Unituxin was approved by FDA in 2015 for the treatment of high-risk neuroblastoma patients. Unituxin (dinutuximab) represents the chimeric GD2-specific Ch14.18 mAb of the IgG1 subtype produced in the SP2/0 cell line. Ch14.18 is also produced in the CHO cell line, which gives a better effector cell response due to the modified glycosylation profile, while preserving the binding properties of the antibody to the ganglioside [216]. The modified antibody, dinutuximab-beta, was also approved by FDA for high-risk neuroblastoma therapy. Ch14.18 displays a significant efficacy in combination therapy and increases the five-year survival rate of neuroblastoma patients by 20%. At the same time, the drug has significant side effects, the main one being neuropathic pain caused by its interaction with sensory neurons [217]. Various immunotherapeutic approaches are being currently developed aimed at improving efficacy and reducing side effects of GD2-specific antibodies [218]. The main clinical trials for neuroblastoma immunotherapy are presented in Table 1.

To date, there are several main trends in the development of GD2-directed immunotherapy:

- Improvement of antitumor effects of GD2-specific antibodies
- Development of immunoconjugates and targeted nanoparticles aimed at GD2-positive tumors
- Use of bispecific antibodies
- Use of adoptive immunotherapy (most importantly, CAR T cells)

#### 3.6.1. Strategies for Improving Antitumor Effects of GD2-Specific Antibodies

The development of GD2-specific monoclonal antibodies for the treatment of GD2-expressing tumors, which, in addition to neuroblastoma, also include melanoma, small cell lung cancer, osteosarcoma, and breast cancer, has been underway for many years [219, 220]. Initially, the use of murine antibodies led to the induction of a human anti-mouse antibody (HAMA) response, which resulted in a faster elimination of antibodies from the body due to neutralizing antibodies. Administration of chimeric antibodies induces human antichimeric antibody (HACA) response, and while the neutralizing antibody titer is significantly lower in this case, it also affects the half-life of antibodies in the body [221].

To reduce the immunogenicity of murine and chimeric GD2-specific antibodies, humanized antibodies Hu3F8 [222] and Hu14.18K322A [223] were created in which only CDRs remained murine, while the rest of the protein was of human origin. Both antibodies showed good results in preclinical trials and in the first phases of clinical trials, demonstrating improved pharmacokinetic characteristics and reduced toxicity [218]. In the case of Hu14.18K322A antibodies which are based on murine GD2-specific antibodies 14.18, reduced side effects and increased efficiency were obtained by modifying the structure of the Fc region and altering antibody glycosylation. A point mutation to lysine 322 allowed to significantly reduce the binding of antibodies to complement proteins and to decrease CDC, which is considered one of the main mechanisms responsible for the side effects of GD2-specific antibodies. ADCC was increased by reducing fucosylation during production of the protein in a YB2/O rat line [224].

A promising approach to improve antitumor effects of GD2-specific antibodies is the use of antibodies to the O-acetylated form of GD2—O-Ac-GD2—in which the outer sialic acid residue is modified with O-acetyl ether [225]. It was shown that O-Ac-GD2 is coexpressed with ganglioside GD2 on tumor cells, and the O-Ac-GD2/GD2 ratio constitutes 10 to 50% [226]. Alvarez-Rueda et al. [227] used monoclonal O-Ac-GD2-specific antibodies 8B6 to show that O-Ac-GD2, unlike GD2, is not present on peripheral neurons and mesenchymal stem cells, as well as on the posterior lobe of the pituitary gland. At the same time, the level of O-Ac-GD2 on GD2-positive tumors remains significant and comparable to that of GD2. The authors demonstrated in a rat model that, in contrast to GD2-specific antibodies ch14.18, chimeric O-Ac-GD2-specific antibodies 8B6 do not induce allodynia [228]. Thus, due to the fact that O-Ac-GD2-specific antibodies lack the main limitation of standard anti-GD2 antibodies which manifests itself in the damage of healthy cells of the body (primarily sensory neurons), O-Ac-GD2 represents a very attractive target for immunotherapy.

Another way to improve GD2-directed therapy is to employ the direct cytotoxic activity of GD2-specific antibodies. Several research groups have shown that a number of GD2-specific antibodies induce cytotoxic effects in tumor cells without involving immune mechanisms [229–231]. Thus, antibodies are capable of triggering morphological changes, cell aggregation and detachment, and inhibition of proliferation by various mechanisms including apoptosis, necrosis, and oncosis-like cell death in different GD2-positive tumor cell lines. The molecular mechanisms of this effect are not completely clear and require additional studies, but the selection of chemotherapy drugs for obtaining a synergistic cytotoxic effect in combination with GD2-specific antibodies is a topical task, which could help to enhance the efficiency of the antitumor therapy [232, 233].

#### 3.6.2. Development of Immunoconjugates and Targeted Nanoparticles Aimed at GD2-Specific Tumors

As part of immunoconjugates or targeted nanoparticles, full-length GD2-specific antibodies and their fragments act as vectors that specifically bind tumor cells. GD2-binding molecules are used to create GD2-directed liposomes [234–236] and nanoparticles [237–240], combinations of anti-GD2 antibodies with chemokines [241], cytokines [242], toxins [243–246], and radioisotopes [199, 247]. Antigen-binding antibody fragments have important advantages for use in immunoconjugates over full-length antibodies and lack a number of their disadvantages, such as poor penetration into solid tumors and Fc-mediated hyperactivation of the immune system [248]. For instance, GD2-specific antibodies and their Fab fragments were covalently attached to stealth liposomes, and the cytotoxic effects of the resulting immunoliposomes loaded with the antitumor drug doxorubicin were assessed in a mouse model of neuroblastoma. Both types of immunoliposomes demonstrated increased cytotoxicity against neuroblastoma cells; however, liposomes conjugated with Fab fragments had a longer circulation time in the blood. [249].

In the case of radioimmunoconjugates, a multistep pretargeted radioimmunotherapy approach that employs GD2-specific antibody-streptavidin constructs is justified. Specifically, 5F11 scFv fragments were fused with streptavidin to form a homotetramer that displayed an increased avidity to GD2; ^111^In-DOTA conjugated to biotin was used as the ligand (^111^In-DOTA-biotin). The results of *in vivo* studies showed a significant increase in the binding of the complex with tumor cells and a decline in the level of nonspecific interactions compared to the single-stage approach using radiolabeled full-length IgG antibodies [250]. In our work, targeted chitosan nanoparticles specific to GD2-positive tumor cells were obtained. Monoclonal GD2-specific antibodies and their Fab and scFv fragments were used as vector molecules, and it was shown that scFv fragments site-specifically conjugated to nanoparticles have a higher antitumor potential compared to full-length antibodies and their Fab fragments [240].

#### 3.6.3. Bispecific Antibodies

Bispecific antibodies (BsAbs) exhibit a dual functionality; i.e., they bind to two different epitopes on the same antigen or on different antigens. BsAbs have significant advantages over conventional antibodies because they can redirect immune effector cells to tumor cells by one of their arms or, alternatively, if both arms are directed to the same tumor cell, inhibit or modulate intracellular signaling pathways more effectively compared to conventional monospecific antibodies [251, 252]. In the case of GD2-positive tumors, the main area of BsAb development is generation of antibodies which bind GD2 with one arm and recruit effector immune cells with the other. In terms of structure, these GD2-specific BsAbs may represent full-length antibodies [253] or antibody fragments [254].

Surek and Ektomab are anti-GD2 trifunctional bispecific full-length antibodies that recruit cytotoxic lymphocytes and target them towards GD2-positive tumors. The authors showed [253] that Surek and Ektomab that contain the Fab fragments to the GD2/GD3-specific antibody ME361 and the T cell-specific CD3 antigen (mouse and human CD3, resp.) demonstrate a pronounced antitumor effect in a mouse melanoma model at lower concentrations than the parent ME361 antibody. They also claim that full-length GD2-specific BsAbs are superior to bispecific antibody fragments; namely, they have a longer circulation time in the blood and also produce a long-term memory response. Disadvantages of these antibodies include low affinity for GD2 (~10^−7^ M), which results in the necessity to increase the therapeutic dose and the preservation of Fc-mediated side effects of parent GD2-specific antibodies. The immunogenicity and specificity of Surek and Ektomab also limit the prospects of their use, since they contain antibody fragments of mouse and rat origin; besides, ME361 antibodies display cross-reactivity to ganglioside GD3, which may increase side effects by influencing healthy body tissues.

BsAbs based on antibody fragments are also being developed for the treatment of neuroblastoma. Cheng et al. [254] produced stable and highly affine bispecific antibodies hu3F8-scBA based on tandem scFv fragments to GD2 and CD3. hu3F8-scBA induced death of GD2-positive tumor cells in femtomolar concentrations and significantly activated T cells *in vitro* and also suppressed tumor growth and prolonged the survival of mice in xenograft models of melanoma and neuroblastoma. This approach also has its limitations, primarily due to the extremely rapid elimination of tandem bispecific antibodies from the body because of their low molecular weight (about 60 kDa) and inability to bind the neonatal FcRn.

An interesting approach that can largely circumvent the limitations of BsAbs in both the standard IgG and tandem scFv format is the use of bispecific IgG-scFv antibodies. The bispecific antibody hu3F8-BsAb consists of a full-length GD2-specific antibody directed to the tumor cell and two CD3-specific scFv fragments linked to the C-terminus of the light chains of the antibody [255]. Hu3F8-BsAb is characterized by high affinity for ganglioside GD2 and a long circulation time in the blood. It demonstrates a high antitumor potential *in vivo*, which results in the effective elimination of the tumor and the absence of the inhibitory effect on effector lymphocytes [256].

#### 3.6.4. CAR T Cells

The generation of CAR T cells is the main direction of adoptive cell therapy for GD2-positive tumors. Human neuroblastoma downregulates MHC class I molecule expression, which represents a major mechanism of tumor escape from the immune control mediated by cytotoxic T lymphocytes. It was also shown that *MYCN* amplification, a well-known bad prognosis factor for neuroblastoma, leads to downmodulation of MHC class I proteins [257]. These facts make CAR T cells which exhibit antitumor activity in an MHC-unrestricted manner a promising approach for this type of cancer.

Ganglioside GD2 is the most exploited target for neuroblastoma therapy with CAR T cells, and most studies use the scFv fragment of GD2-specific antibodies as the antigen-recognition part of CAR T cells [55]. The results of application of first-generation CAR T cells targeted to GD2 on neuroblastoma cells gave optimistic results [258, 259]. GD2-directed CAR T cells demonstrated good tolerability in phase I clinical studies, as well as absence of neuropathic pain, a common side effect of GD2-specific antibodies, but the detailed information about the antitumor activity was not provided [260, 261].

Later, researchers that developed the next generations of GD2-specific CAR T cells encountered difficulties and limitations that are typical for chimeric antigen receptor T cells targeting all types of solid tumors. Several of the most important limitations that hinder the development of CAR T therapies for solid tumors are as follows:

- Cell and antigen heterogeneity in solid tumors [262]
- On-target/off-tumor toxicity caused by expression of targets for CAR T cells on healthy body cells
- The inhibitory effect of the tumor microenvironment of solid tumors, and the limited ability of CAR T cells to penetrate solid tumors due to physical barriers from the surrounding stroma and infiltrating protumor immune cells, as well as absence of chemokine receptors [262, 263],
- Suppression of T cell functional activity, which manifests itself in early T cell exhaustion due to tonic signaling, and activation-induced cell death (AICD) of CAR T cells upon interaction with the antigen [263, 264]

If these limitations are not overcome, the tremendous success of CAR T cell adoptive immunotherapies in eradicating hematological malignancies will likely not be extrapolated to solid tumors and, specifically, to neuroblastoma [265]. A variety of approaches are currently being developed to overcome the aforementioned limitations, including, among others, the introduction of costimulatory molecules (primarily 4-1BB) into the intracellular domain of the receptor, blockade of checkpoint inhibitors (e.g., PD-1), constitutive activation of proliferative and inhibition of apoptotic signal pathways in CAR T cells, and modification of the structure of the chimeric antigen receptor and its density on the surface of T cells [264, 266–268].

Limitations in the use of anti-GD2 CAR T therapies are yet to be overcome. Hoseini et al. [256] made an attempt to identify therapeutic barriers facing CAR-modified GD2-specific T cells by comparing their antitumor effects with T cells redirected by anti-GD2 bispecific antibodies in the IgG-scFv format mentioned earlier on a GD2-positive xenograft model of melanoma. For this, second-generation anti-GD2 CAR T cells with different receptor affinities were generated. The viability of CAR T cells after encountering effector cells was inferior to that of the T cells that interacted with melanoma via bispecific anti-GD2/CD3 antibodies. The authors demonstrated that AICD of CAR T cells depends on the density of the receptor on the cell surface: T cells with high receptor density died when they encountered effector cells, whereas those with low receptor density remained viable. The inclusion of the costimulatory domain 4-1BB, the affinity of the receptor, and blocking PD-1 did not decrease cell depletion of GD2-specific CARTs, and their exhaustion remained significant in all cases.

Current clinical trials employing GD2-specific CAR T cells for the treatment of neuroblastoma investigate CARTs that contain a suicide gene safety switch (most often, inducible сaspase 9), which is used to mitigate the risks of toxicity either from cytokine release syndrome or from on-target/off-tumor effects on healthy tissues. Specifically, a third-generation GD2-specific iC9 CD28.OX40.*ζ* CAR investigated in a phase I study in combination with lymphodepletion and PD-1 inhibition was safe and showed no dose-limiting toxicities; however, the antitumor response was modest, and PD-1 inhibition did not further enhance T cell expansion or persistence [269, 270]. Thus, employing CARTs to direct T cell immunity against GD2-positive tumor cells is potentially a very effective approach, but additional research in this topic is required to justify the application of this immunotherapy platform in neuroblastoma therapy.

## 4. Summary

Improved early detection and use of immunotherapy have led to a relative success in the treatment of neuroblastoma. Unituxin, a chimeric GD2-specific antibody, demonstrates efficacy in increasing survival rates in patients receiving standard multimodal therapy and is becoming a standard component of combination therapy for HR-NB. An important characteristic of this immunotherapeutic approach is that it has no long-term and cumulative toxicity, which is especially important for the child's organism. At the same time, immunotherapy with GD2-specific antibodies cannot be considered optimal, since it has pronounced side effects primarily associated with on-target/off-tumor toxicity. In most patients (~85%) taking Unituxin, severe pain is observed which may be removed only by potent analgesics. Other side effects common for this medication include pyrexia, hypotension, and capillary leak syndrome [5]. Therefore, effective HR-NB immunotherapy requires the optimization of existing and the development of novel approaches.

Strategies to improve the therapeutic properties of GD2-specific antibodies consist in their humanization and modification by point mutations aimed at reducing the immunogenicity and side effects of antibodies. Clinical application of monoclonal antibodies specific to the O-acetylated form of ganglioside GD2 may help to reduce on-target/off-tumor effects caused primarily by the action of GD2-specific antibodies on sensory neurons; expression of O-Ac-GD2, unlike GD2, has so far not been shown on sensory neurons, mesenchymal stromal cells, and the posterior lobe of the pituitary gland. Another important area for improvement of HR-NB therapy with Unituxin is the adjustment of drug dosage and optimal combination therapies with chemotherapy drugs and cytokines. Selection of chemotherapy regimens for obtaining a synergistic antitumor effect in combination with antibodies is a topical task. For instance, the combination of irinotecan and temozolomide with Unituxin gives a synergistic antitumor effect compared to separate use of these medications. The molecular mechanisms of this effect are not completely clear and require additional studies. However, the high heterogeneity of neuroblastoma will likely require a personalized approach with an individual assessment of the genetic, biochemical, and phenotypic features of neuroblastoma cells of each particular patient to select optimal combination therapies.

Modern immunotherapy strategies such as immune checkpoint inhibitors and CAR T cells, which led to revolutionary advances in the therapy of a number of cancers, have demonstrated limited progress in neuroblastoma treatment. The very low levels of HLA class I and PD-1 molecules on neuroblastoma cells do not allow to obtain significant antitumor responses with checkpoint inhibitors. Also, CAR T cells directed to neuroblastoma cells demonstrate a much lower efficiency in eliminating tumor cells compared to CD19-specific CAR T cells in the therapy of leukemia. The problem of rapid exhaustion of CAR T cells targeted to solid tumors, including neuroblastoma, that leads to low antitumor activity has not been solved yet. Using bispecific antibodies that redirect immune effector cells to neuroblastoma cells is a potentially promising technology, but it is still on an early stage of development.

High-risk neuroblastoma is a heterogeneous cancer that has a poor response to treatment. Development of effective immunotherapeutic approaches for the therapy of HR-NB will require a deep understanding of the biological characteristics of its development, a comprehensive study of known molecular markers and search for novel markers on neuroblastoma cells, use of a personalized approach to assess the genetic profile and specifics of the signaling pathways of each patient, as well as adaptation of modern immunotherapeutic platforms for the therapy of neuroblastoma.

## Figures and Tables

**Table 1:** Currently active clinical trials for neuroblastoma immunotherapy.

Anti-GD2 immunotherapy				
Approach	Agent combination	Phase	NCT identifier	Start year
Naked antibody (murine 3F8)	3F8, allogeneic NK cells, cyclophosphamide, vincristine, topotecan	I	NCT00877110 ^∗^	2009
	3F8, GM-CSF, isotretinoin	II	NCT01183897 ^∗^, NCT01183884^∗^	2010
		N/A	NCT02100930	2014

Naked antibody (chimeric dinutuximab)	Dinutuximab, irinotecan, temozolomide	II	NCT01767194 ^∗^	2013
	Dinutuximab, autologous NK cells, lenalidomide	I	NCT02573896	2015
	Dinutuximab, ^131^I-metaiodobenzylguanidine	I	NCT03332667	2017

Naked antibody (chimeric dinutuximab-beta, produced in CHO)	Dinutuximab-beta, IL-2, isotretinoin	III	NCT01704716	2012
	Dinutuximab-beta	II	NCT02743429	2016
	Dinutuximab-beta, nivolumab	I	NCT02914405	2016
	Dinutuximab-beta, haploidentical NK cells, IL-2, cyclophosphamide	I/II	NCT03242603	2017

Naked antibody (humanized hu3F8)	hu3F8	I	NCT01419834	2011
	hu3F8, GM-CSF	I/II	NCT01757626 ^∗^	2012
		III	NCT03363373	2017
	hu3F8, IL-2	I	NCT01662804 ^∗^	2012
	hu3F8, haploidentical NK cells, IL-2, cyclophosphamide	I	NCT02650648	2016
	hu3F8, GM-CSF, isotretinoin	II	NCT03033303	2017
	hu3F8, irinotecan, temozolomide, GM-CSF	Pilot	NCT03189706	2017

Naked antibody (humanized hu14.18)	hu14.18K322A, allogeneic NK cells, IL-2, GM-CSF, combination chemotherapy	I	NCT01576692 ^∗^	2012
	hu14.18K322A, allogeneic NK cells, IL-2, G-CSF, GM-CSF, combination chemotherapy	II	NCT01857934	2013

Immunocytokine	hu14.18-IL2, haploidentical NK cells	I	NCT03209869	2017

Radioimmunoconjugate	^131^I-3F8	II	NCT00445965 ^∗^	2007

T cell engaging bispecific antibody	hu3F8-scBA, activated T cells, GM-CSF, IL-2	I/II	NCT02173093	2014

Adoptive NK cell transfer	HLA-haploidentical HCT, allogeneic NK cells	II	NCT02100891	2014

Adoptive NKT cell transfer	Autologous anti-GD2 NKT cells expressing IL-15, cyclophosphamide, fludarabine	I	NCT03294954	2017

Adoptive T cell transfer	Autologous anti-GD2 3rd-gen iC9 CAR-modified T cells, pembrolizumab, cyclophosphamide, fludarabine	I	NCT01822652 ^∗^	2013
	Autologous anti-GD2 4th-gen iC9 CAR-modified T cells	II	NCT02765243	2016
	Autologous anti-GD2 2nd-gen CAR-modified T cells, cyclophosphamide, fludarabine	I	NCT02761915	2016
	Autologous anti-GD2 iC9 CAR-modified T cells	I/II	NCT03373097	2017

Vaccine	Gene-modified SJNB-JF-IL2 and SJNB-JF-LTN in conjunction with unmodified SKNLP (neuroblastoma cell vaccine)	I/II	NCT00703222 ^∗^	2008
	Bivalent GD2/GD3 lactone vaccine with OPT-821 adjuvant, oral *β*-glucan	I/II	NCT00911560	2009
	Gene-modified SJNB-JF-IL2 and SJNB-JF-LTN in conjunction with unmodified SKNLP (neuroblastoma cell vaccine), oral cyclophosphamide	I/II	NCT01192555 ^∗^	2010

*Anti-CD171 immunotherapy*				

Adoptive T cell transfer	Autologous anti-CD171 CAR-modified T cells (2^nd^- and 3rd-gen) expressing EGFRt	I	NCT02311621	2014

*Anti-NGcGM3 immunotherapy*				

Naked antibody (anti-idiotype vaccine, murine)	Racotumomab	II	NCT02998983	2016

*Anti-B7-H3 immunotherapy*				

Radioimmunotherapy (murine ^131^I-8H9)	^131^I-conjugated omburtomab	I	NCT00089245	2004
	^131^I-conjugated omburtomab	II/III	NCT03275402	2017

*Anti-NY-ESO-1 immunotherapy*				

Adoptive T cell transfer	Autologous anti-NY-ESO-1 CAR-modified T cells	I	NCT02457650	2015

^∗^Active, not recruiting (from http://clinicaltrials.gov). CAR: chimeric antigen receptor; EGFRt: truncated epidermal growth factor receptor; HCT: hematopoietic cell transplantation; HLA: human leukocyte antigen; hu3F8-scBA: anti-GD2/anti-CD3 (hu3F8): single chain bispecific antibody; iC9: inducible caspase 9 suicide gene.

## References

[1] Maris J. M., Hogarty M. D., Bagatell R., Cohn S. L. (2007). Neuroblastoma. *Lancet*.

[2] Whittle S. B., Smith V., Doherty E., Zhao S., McCarty S., Zage P. E. (2017). Overview and recent advances in the treatment of neuroblastoma. *Expert Review of Anticancer Therapy*.

[3] Arakawa A., Oguma E., Aihara T. (2014). Long-term follow-up results of the observation program for neuroblastoma detected at 6-month mass screening. *The Journal of Pediatrics*.

[4] Yu A. L., Gilman A. L., Ozkaynak M. F. (2010). Anti-GD2 antibody with GM-CSF, interleukin-2, and isotretinoin for neuroblastoma. *The New England Journal of Medicine*.

[5] McGinty L., Kolesar J. (2017). Dinutuximab for maintenance therapy in pediatric neuroblastoma. *American Journal of Health-System Pharmacy*.

[6] Simoes-Costa M., Bronner M. E. (2013). Insights into neural crest development and evolution from genomic analysis. *Genome Research*.

[7] Bronner M. E., Simoes-Costa M. (2016). The neural crest migrating into the twenty-first century. *Current Topics in Developmental Biology*.

[8] Simoes-Costa M., Bronner M. E. (2015). Establishing neural crest identity: a gene regulatory recipe. *Development*.

[9] Motohashi T., Kunisada T. (2015). Extended multipotency of neural crest cells and neural crest-derived cells. *Current Topics in Developmental Biology*.

[10] Toma J. G., McKenzie I. A., Bagli D., Miller F. D. (2005). Isolation and characterization of multipotent skin-derived precursors from human skin. *Stem Cells*.

[11] Johnston A. P. W., Naska S., Jones K., Jinno H., Kaplan D. R., Miller F. D. (2013). Sox2-mediated regulation of adult neural crest precursors and skin repair. *Stem Cell Reports*.

[12] Li H. Y., Say E. H. M., Zhou X. F. (2007). Isolation and characterization of neural crest progenitors from adult dorsal root ganglia. *Stem Cells*.

[13] Chung K. F., Sicard F., Vukicevic V. (2009). Isolation of neural crest derived chromaffin progenitors from adult adrenal medulla. *Stem Cells*.

[14] Nagoshi N., Shibata S., Kubota Y. (2008). Ontogeny and multipotency of neural crest-derived stem cells in mouse bone marrow, dorsal root ganglia, and whisker pad. *Cell Stem Cell*.

[15] Shakhova O. (2014). Neural crest stem cells in melanoma development. *Current Opinion in Oncology*.

[16] Sieber-Blum M., Hu Y. (2008). Mouse epidermal neural crest stem cell (EPI-NCSC) cultures. *Journal of Visualized Experiments*.

[17] Achilleos A., Trainor P. A. (2012). Neural crest stem cells: discovery, properties and potential for therapy. *Cell Research*.

[18] Zage P. E., Whittle S. B., Shohet J. M. (2017). CD114: a new member of the neural crest-derived cancer stem cell marker family. *Journal of Cellular Biochemistry*.

[19] Gupta P. B., Kuperwasser C., Brunet J. P. (2005). The melanocyte differentiation program predisposes to metastasis after neoplastic transformation. *Nature Genetics*.

[20] Bailey C. M., Morrison J. A., Kulesa P. M. (2012). Melanoma revives an embryonic migration program to promote plasticity and invasion. *Pigment Cell & Melanoma Research*.

[21] Barrallo-Gimeno A., Nieto M. A. (2005). The Snail genes as inducers of cell movement and survival: implications in development and cancer. *Development*.

[22] Moreno-Bueno G., Portillo F., Cano A. (2008). Transcriptional regulation of cell polarity in EMT and cancer. *Oncogene*.

[23] Theveneau E., Mayor R. (2012). Neural crest delamination and migration: from epithelium-to-mesenchyme transition to collective cell migration. *Developmental Biology*.

[24] Matthay K. K., Maris J. M., Schleiermacher G. (2016). Neuroblastoma. *Nature Reviews Disease Primers*.

[25] Anderson D. J. (1993). Molecular control of cell fate in the neural crest: the sympathoadrenal lineage. *Annual Review of Neuroscience*.

[26] Howard M. J., Stanke M., Schneider C., Wu X., Rohrer H. (2000). The transcription factor dHAND is a downstream effector of BMPs in sympathetic neuron specification. *Development*.

[27] Ernsberger U., Esposito L., Partimo S. (2005). Expression of neuronal markers suggests heterogeneity of chick sympathoadrenal cells prior to invasion of the adrenal anlagen. *Cell and Tissue Research*.

[28] Reissmann E., Ernsberger U., Francis-West P. H., Rueger D., Brickell P. M., Rohrer H. (1996). Involvement of bone morphogenetic protein-4 and bone morphogenetic protein-7 in the differentiation of the adrenergic phenotype in developing sympathetic neurons. *Development*.

[29] Schneider C., Wicht H., Enderich J., Wegner M., Rohrer H. (1999). Bone morphogenetic proteins are required *in vivo* for the generation of sympathetic neurons. *Neuron*.

[30] Huber K., Franke A., Brühl B. (2008). Persistent expression of BMP-4 in embryonic chick adrenal cortical cells and its role in chromaffin cell development. *Neural Development*.

[31] Betters E., Liu Y., Kjaeldgaard A., Sundstrom E., Garcia-Castro M. I. (2010). Analysis of early human neural crest development. *Developmental Biology*.

[32] Huber K. (2006). The sympathoadrenal cell lineage: specification, diversification, and new perspectives. *Developmental Biology*.

[33] Yuan J., Yankner B. A. (2000). Apoptosis in the nervous system. *Nature*.

[34] Marshall G. M., Carter D. R., Cheung B. B. (2014). The prenatal origins of cancer. *Nature Reviews Cancer*.

[35] Brodeur G. M. (2003). Neuroblastoma: biological insights into a clinical enigma. *Nature Reviews Cancer*.

[36] Maris J. M. (2010). Recent advances in neuroblastoma. *The New England Journal of Medicine*.

[37] Beckwith J. B., Perrin E. V. (1963). In situ neuroblastomas: a contribution to the natural history of neural crest tumors. *The American Journal of Pathology*.

[38] Weiss W. A., Aldape K., Mohapatra G., Feuerstein B. G., Bishop J. M. (1997). Targeted expression of *MYCN* causes neuroblastoma in transgenic mice. *The EMBO Journal*.

[39] Bailey C. M., Kulesa P. M. (2014). Dynamic interactions between cancer cells and the embryonic microenvironment regulate cell invasion and reveal EphB6 as a metastasis suppressor. *Molecular Cancer Research*.

[40] de Preter K., Vandesompele J., Heimann P. (2006). Human fetal neuroblast and neuroblastoma transcriptome analysis confirms neuroblast origin and highlights neuroblastoma candidate genes. *Genome Biology*.

[41] Furlan A., Dyachuk V., Kastriti M. E. (2017). Multipotent peripheral glial cells generate neuroendocrine cells of the adrenal medulla. *Science*.

[42] Stine Z. E., Walton Z. E., Altman B. J., Hsieh A. L., Dang C. V. (2015). MYC, metabolism, and cancer. *Cancer Discovery*.

[43] Kerosuo L., Bronner M. E. (2016). cMyc regulates the size of the premigratory neural crest stem cell pool. *Cell Reports*.

[44] Bellmeyer A., Krase J., Lindgren J., LaBonne C. (2003). The protooncogene c-myc is an essential regulator of neural crest formation in *xenopus*. *Developmental Cell*.

[45] Huang M., Weiss W. A. (2013). Neuroblastoma and MYCN. *Cold Spring Harbor Perspectives in Medicine*.

[46] Wartiovaara K., Barnabe-Heider F., Miller F. D., Kaplan D. R. (2002). N-myc promotes survival and induces S-phase entry of postmitotic sympathetic neurons. *The Journal of Neuroscience*.

[47] Hansford L. M., Thomas W. D., Keating J. M. (2004). Mechanisms of embryonal tumor initiation: distinct roles for MycN expression and MYCN amplification. *Proceedings of the National Academy of Sciences of the United States of America*.

[48] Kramer M., Ribeiro D., Arsenian-Henriksson M., Deller T., Rohrer H. (2016). Proliferation and survival of embryonic sympathetic neuroblasts by MYCN and activated ALK signaling. *The Journal of Neuroscience*.

[49] Zhang J. T., Weng Z. H., Tsang K. S., Tsang L. L., Chan H. C., Jiang X. H. (2016). MycN is critical for the maintenance of human embryonic stem cell-derived neural crest stem cells. *PLoS One*.

[50] Westermark U. K., Wilhelm M., Frenzel A., Henriksson M. A. (2011). The MYCN oncogene and differentiation in neuroblastoma. *Seminars in Cancer Biology*.

[51] Zhu S., Lee J. S., Guo F. (2012). Activated ALK collaborates with MYCN in neuroblastoma pathogenesis. *Cancer Cell*.

[52] Schulte J. H., Lindner S., Bohrer A. (2013). MYCN and ALKF1174L are sufficient to drive neuroblastoma development from neural crest progenitor cells. *Oncogene*.

[53] Olsen R. R., Otero J. H., García-López J. (2017). MYCN induces neuroblastoma in primary neural crest cells. *Oncogene*.

[54] Azarova A. M., Gautam G., George R. E. (2011). Emerging importance of ALK in neuroblastoma. *Seminars in Cancer Biology*.

[55] Cheung N. K. V., Dyer M. A. (2013). Neuroblastoma: developmental biology, cancer genomics and immunotherapy. *Nature Reviews Cancer*.

[56] Reiff T., Huber L., Kramer M., Delattre O., Janoueix-Lerosey I., Rohrer H. (2011). Midkine and Alk signaling in sympathetic neuron proliferation and neuroblastoma predisposition. *Development*.

[57] Hallberg B., Palmer R. H. (2013). Mechanistic insight into ALK receptor tyrosine kinase in human cancer biology. *Nature Reviews Cancer*.

[58] Cheng L. Y., Bailey A. P., Leevers S. J., Ragan T. J., Driscoll P. C., Gould A. P. (2011). Anaplastic lymphoma kinase spares organ growth during nutrient restriction in *Drosophila*. *Cell*.

[59] Yao S., Cheng M., Zhang Q., Wasik M., Kelsh R., Winkler C. (2013). Anaplastic lymphoma kinase is required for neurogenesis in the developing central nervous system of zebrafish. *PLoS One*.

[60] Duijkers F. A. M., Gaal J., Meijerink J. P. P. (2012). High anaplastic lymphoma kinase immunohistochemical staining in neuroblastoma and ganglioneuroblastoma is an independent predictor of poor outcome. *The American Journal of Pathology*.

[61] Wang M., Zhou C., Sun Q. (2013). *ALK* amplification and protein expression predict inferior prognosis in neuroblastomas. *Experimental and Molecular Pathology*.

[62] De Brouwer S., De Preter K., Kumps C. (2010). Meta-analysis of neuroblastomas reveals a skewed *ALK* mutation spectrum in tumors with *MYCN* amplification. *Clinical Cancer Research*.

[63] Passoni L., Longo L., Collini P. (2009). Mutation independent anaplastic lymphoma kinase overexpression in poor prognosis neuroblastoma patients. *Cancer Research*.

[64] Schulte J. H., Bachmann H. S., Brockmeyer B. (2011). High *ALK* receptor tyrosine kinase expression supersedes *ALK* mutation as a determining factor of an unfavorable phenotype in primary neuroblastoma. *Clinical Cancer Research*.

[65] Montavon G., Jauquier N., Coulon A. (2014). Wild-type ALK and activating ALK-R1275Q and ALK-F1174L mutations upregulate Myc and initiate tumor formation in murine neural crest progenitor cells. *Oncotarget*.

[66] Pandian V., Ramraj S., Khan F. H., Azim T., Aravindan N. (2015). Metastatic neuroblastoma cancer stem cells exhibit flexible plasticity and adaptive stemness signaling. *Stem Cell Research & Therapy*.

[67] Shimada H., Umehara S., Monobe Y. (2001). International neuroblastoma pathology classification for prognostic evaluation of patients with peripheral neuroblastic tumors. *Cancer*.

[68] van Groningen T., Koster J., Valentijn L. J. (2017). Neuroblastoma is composed of two super-enhancer-associated differentiation states. *Nature Genetics*.

[69] Boeva V., Louis-Brennetot C., Peltier A. (2017). Heterogeneity of neuroblastoma cell identity defined by transcriptional circuitries. *Nature Genetics*.

[70] Rettig W. J., Spengler B. A., Chesa P. G., Old L. J., Biedler J. L. (1987). Coordinate changes in neuronal phenotype and surface antigen expression in human neuroblastoma cell variants. *Cancer Research*.

[71] Walton J. D., Kattan D. R., Thomas S. K. (2004). Characteristics of stem cells from human neuroblastoma cell lines and in tumors. *Neoplasia*.

[72] Ross R. A., Biedler J. L., Spengler B. A. (2003). A role for distinct cell types in determining malignancy in human neuroblastoma cell lines and tumors. *Cancer Letters*.

[73] Ross R. A., Spengler B. A., Domenech C., Porubcin M., Rettig W. J., Biedler J. L. (1995). Human neuroblastoma I-type cells are malignant neural crest stem cells. *Cell Growth & Differentiation*.

[74] Ross R. A., Spengler B. A. (2007). Human neuroblastoma stem cells. *Seminars in Cancer Biology*.

[75] Ross R. A., Walton J. D., Han D., Guo H. F., Cheung N. K. V. (2015). A distinct gene expression signature characterizes human neuroblastoma cancer stem cells. *Stem Cell Research*.

[76] Hirschmann-Jax C., Foster A. E., Wulf G. G. (2004). A distinct “side population” of cells with high drug efflux capacity in human tumor cells. *Proceedings of the National Academy of Sciences of the United States of America*.

[77] Westover D., Li F. (2015). New trends for overcoming ABCG2/BCRP-mediated resistance to cancer therapies. *Journal of Experimental & Clinical Cancer Research*.

[78] Rahgozar S., Moafi A., Abedi M. (2013). mRNA expression profile of multidrug-resistant genes in acute lymphoblastic leukemia of children, a prognostic value for ABCA3 and ABCA2. *Cancer Biology & Therapy*.

[79] Kreso A., Dick J. E. (2014). Evolution of the cancer stem cell model. *Cell Stem Cell*.

[80] Geissler E. N., Ryan M. A., Housman D. E. (1988). The dominant white spotting (*w*) locus of the mouse encodes the c-kit proto-oncogene. *Cell*.

[81] Rubin B. P., Singer S., Tsao C. (2001). KIT activation is a ubiquitous feature of gastrointestinal stromal tumors. *Cancer Research*.

[82] Longley B. J., Metcalfe D. D., Tharp M. (1999). Activating and dominant inactivating c-KIT catalytic domain mutations in distinct clinical forms of human mastocytosis. *Proceedings of the National Academy of Sciences of the United States of America*.

[83] Meshinchi S., Stirewalt D. L., Alonzo T. A. (2003). Activating mutations of RTK/ras signal transduction pathway in pediatric acute myeloid leukemia. *Blood*.

[84] Kemmer K., Corless C. L., Fletcher J. A. (2004). KIT mutations are common in testicular seminomas. *The American Journal of Pathology*.

[85] Rygaard K., Nakamura T., Spang-Thomsen M. (1993). Expression of the proto-oncogenes c-*met* and c-*kit* and their ligands, hepatocyte growth factor/scatter factor and stem cell factor, in SCLC cell lines and xenografts. *British Journal of Cancer*.

[86] Cohen P. S., Chan J. P., Lipkunskaya M., Biedler J. L., Seeger R. C., The children’s cancer group (1994). Expression of stem cell factor and c-*kit* in human neuroblastoma. *Blood*.

[87] Bellone G., Silvestri S., Artusio E. (1997). Growth stimulation of colorectal carcinoma cells via the c-kit receptor is inhibited by TGF-*β*1. *Journal of Cellular Physiology*.

[88] Tonary A. M., Macdonald E. A., Faught W., Senterman M. K., Vanderhyden B. C. (2000). Lack of expression of c-*KIT* in ovarian cancers is associated with poor prognosis. *International Journal of Cancer*.

[89] Beck D., Gross N., Brognara C. B., Perruisseau G. (1995). Expression of stem cell factor and its receptor by human neuroblastoma cells and tumors. *Blood*.

[90] Krams M., Parwaresch R., Sipos B., Heidorn K., Harms D., Rudolph P. (2004). Expression of the c-kit receptor characterizes a subset of neuroblastomas with favorable prognosis. *Oncogene*.

[91] Uccini S., Mannarino O., McDowell H. P. (2005). Clinical and molecular evidence for c-kit receptor as a therapeutic target in neuroblastic tumors. *Clinical Cancer Research*.

[92] Lebedev T. D., Spirin P. V., Orlova N. N., Prokofjeva M. M., Prassolov V. S. (2015). Comparative analysis of gene expression: targeted antitumor therapy in neuroblastoma cell lines. *Molecular Biology*.

[93] Vitali R., Cesi V., Nicotra M. R. (2003). C-kit is preferentially expressed in *MYCN*-amplified neuroblastoma and its effect on cell proliferation is inhibited *in vitro* by STI-571. *International Journal of Cancer*.

[94] Lupino E., Ramondetti C., Buccinna B., Piccinini M. (2014). Exposure of neuroblastoma cell lines to imatinib results in the upregulation of the CDK inhibitor p27^KIP1^ as a consequence of c-Abl inhibition. *Biochemical Pharmacology*.

[95] Beppu K., Jaboine J., Merchant M. S., Mackall C. L., Thiele C. J. (2004). Effect of imatinib mesylate on neuroblastoma tumorigenesis and vascular endothelial growth factor expression. *Journal of the National Cancer Institute*.

[96] Lebedev T. D., Spirin P. V., Suntsova M. V. (2015). Receptor tyrosine kinase KIT can regulate the expression of genes involved in spontaneous regression of neuroblastoma. *Molecular Biology*.

[97] Nishida T., Blay J. Y., Hirota S., Kitagawa Y., Kang Y. K. (2016). The standard diagnosis, treatment, and follow-up of gastrointestinal stromal tumors based on guidelines. *Gastric Cancer*.

[98] Rosenzweig S. A. (2012). Acquired resistance to drugs targeting receptor tyrosine kinases. *Biochemical Pharmacology*.

[99] Kato M., Takeda K., Kawamoto Y. (2004). C-kit-targeting immunotherapy for hereditary melanoma in a mouse model. *Cancer Research*.

[100] London C. A., Gardner H. L., Rippy S. (2017). KTN0158, a humanized anti-KIT monoclonal antibody, demonstrates biologic activity against both normal and malignant canine mast cells. *Clinical Cancer Research*.

[101] Stahl M., Gedrich R., Peck R., LaVallee T., Eder J. P. (2016). Targeting KIT on innate immune cells to enhance the antitumor activity of checkpoint inhibitors. *Immunotherapy*.

[102] Garton A. J., Seibel S., Lopresti-Morrow L. (2017). Anti-KIT monoclonal antibody treatment enhances the antitumor activity of immune checkpoint inhibitors by reversing tumor-induced immunosuppression. *Molecular Cancer Therapeutics*.

[103] Wognum A. W., Eaves A. C., Thomas T. E. (2003). Identification and isolation of hematopoietic stem cells. *Archives of Medical Research*.

[104] Bhatia M. (2001). AC133 expression in human stem cells. *Leukemia*.

[105] Horst D., Kriegl L., Engel J., Kirchner T., Jung A. (2008). CD133 expression is an independent prognostic marker for low survival in colorectal cancer. *British Journal of Cancer*.

[106] Uchida N., Buck D. W., He D. (2000). Direct isolation of human central nervous system stem cells. *Proceedings of the National Academy of Sciences of the United States of America*.

[107] Li Z. (2013). CD133: a stem cell biomarker and beyond. *Experimental Hematology & Oncology*.

[108] Yu X., Lin Y., Yan X., Tian Q., Li L., Lin E. H. (2011). CD133, stem cells, and cancer stem cells: myth or reality?. *Current Colorectal Cancer Reports*.

[109] Chen Y. L., Lin P. Y., Ming Y. Z. (2017). The effects of the location of cancer stem cell marker CD133 on the prognosis of hepatocellular carcinoma patients. *BMC Cancer*.

[110] Zeppernick F., Ahmadi R., Campos B. (2008). Stem cell marker CD133 affects clinical outcome in glioma patients. *Clinical Cancer Research*.

[111] Luo R., Gao J., Wehrle-Haller B., Henion P. D. (2003). Molecular identification of distinct neurogenic and melanogenic neural crest sublineages. *Development*.

[112] Tong Q. S., Zheng L. D., Tang S. T. (2008). Expression and clinical significance of stem cell marker CD133 in human neuroblastoma. *World Journal of Pediatrics*.

[113] Sartelet H., Imbriglio T., Nyalendo C. (2012). CD133 expression is associated with poor outcome in neuroblastoma via chemoresistance mediated by the AKT pathway. *Histopathology*.

[114] Takenobu H., Shimozato O., Nakamura T. (2011). CD133 suppresses neuroblastoma cell differentiation via signal pathway modification. *Oncogene*.

[115] Mehrazma M., Madjd Z., Kalantari E., Panahi M., Hendi A., Shariftabrizi A. (2012). Expression of stem cell markers, CD133 and CD44, in pediatric solid tumors: a study using tissue microarray. *Fetal and Pediatric Pathology*.

[116] de la Rosa J., Asensio-Salazar J., Shahi M. H. (2017). Genetic and functional characterization of cyclopamine resistant neuroblastoma cells. *Acta Scientific Cancer Biology*.

[117] Zhong Z. Y., Shi B. J., Zhou H., Wang W. B. (2018). CD133 expression and *MYCN* amplification induce chemoresistance and reduce average survival time in pediatric neuroblastoma. *The Journal of International Medical Research*.

[118] Waldron N. N., Kaufman D. S., Oh S. (2011). Targeting tumor-initiating cancer cells with dCD133KDEL shows impressive tumor reductions in a xenotransplant model of human head and neck cancer. *Molecular Cancer Therapeutics*.

[119] Ohlfest J. R., Zellmer D. M., Panyam J. (2013). Immunotoxin targeting CD133^+^ breast carcinoma cells. *Drug Delivery and Translational Research*.

[120] Skubitz A. P. N., Taras E. P., Boylan K. L. M. (2013). Targeting CD133 in an in vivo ovarian cancer model reduces ovarian cancer progression. *Gynecologic Oncology*.

[121] Swaminathan S. K., Roger E., Toti U., Niu L., Ohlfest J. R., Panyam J. (2013). CD133-targeted paclitaxel delivery inhibits local tumor recurrence in a mouse model of breast cancer. *Journal of Controlled Release*.

[122] Stratford E. W., Bostad M., Castro R. (2013). Photochemical internalization of CD133-targeting immunotoxins efficiently depletes sarcoma cells with stem-like properties and reduces tumorigenicity. *Biochimica et Biophysica Acta*.

[123] Huang J., Li C., Wang Y. (2013). Cytokine-induced killer (CIK) cells bound with anti-CD3/anti-CD133 bispecific antibodies target CD133^high^ cancer stem cells in vitro and in vivo. *Clinical Immunology*.

[124] Schmohl J. U., Gleason M. K., Dougherty P. R., Miller J. S., Vallera D. A. (2016). Heterodimeric bispecific single chain variable fragments (scFv) killer engagers (BiKEs) enhance NK-cell activity against CD133+ colorectal cancer cells. *Targeted Oncology*.

[125] Zhu X., Prasad S., Gaedicke S., Hettich M., Firat E., Niedermann G. (2015). Patient-derived glioblastoma stem cells are killed by CD133-specific CAR T cells but induce the T cell aging marker CD57. *Oncotarget*.

[126] Hsu D. M., Agarwal S., Benham A. (2013). G-CSF receptor positive neuroblastoma subpopulations are enriched in chemotherapy-resistant or relapsed tumors and are highly tumorigenic. *Cancer Research*.

[127] Agarwal S., Lakoma A., Chen Z. (2015). G-CSF promotes neuroblastoma tumorigenicity and metastasis via STAT3-dependent cancer stem cell activation. *Cancer Research*.

[128] Zhang L., Agarwal S., Shohet J. M., Zage P. E. (2015). CD114 expression mediates melanoma tumor cell growth and treatment resistance. *Anticancer Research*.

[129] Yan B., Wei J. J., Yuan Y. (2013). IL-6 cooperates with G-CSF to induce protumor function of neutrophils in bone marrow by enhancing STAT3 activation. *Journal of Immunology*.

[130] Chakraborty A., Guha S. (2007). Granulocyte colony-stimulating factor/granulocyte colony-stimulating factor receptor biological axis promotes survival and growth of bladder cancer cells. *Urology*.

[131] Russell H., Shohet J. M. (2011). Pediatric oncology: G-CSF counteracts chemotherapy toxicity in neuroblastoma. *Nature Reviews. Clinical Oncology*.

[132] Bronner-Fraser M. (1986). Analysis of the early stages of trunk neural crest migration in avian embryos using monoclonal antibody HNK-1. *Developmental Biology*.

[133] Bronner-Fraser M. (1987). Perturbation of cranial neural crest migration by the HNK-1 antibody. *Developmental Biology*.

[134] Thies A., Schachner M., Berger J. (2004). The developmentally regulated neural crest-associated glycotope HNK-1 predicts metastasis in cutaneous malignant melanoma. *The Journal of Pathology*.

[135] Bozzi F., Collini P., Aiello A. (2008). Flow cytometric phenotype of rhabdomyosarcoma bone marrow metastatic cells and its implication in differential diagnosis with neuroblastoma. *Anticancer Research*.

[136] Hata J. L., Correa H., Krishnan C. (2015). Diagnostic utility of PHOX2B in primary and treated neuroblastoma and in neuroblastoma metastatic to the bone marrow. *Archives of Pathology & Laboratory Medicine*.

[137] Schlitter A. M., Dorneburg C., Barth T. F. E. (2012). CD57^high^ neuroblastoma cells have aggressive attributes *ex situ* and an undifferentiated phenotype in patients. *PLoS One*.

[138] Rathjen F. G., Schachner M. (1984). Immunocytological and biochemical characterization of a new neuronal cell surface component (L1 antigen) which is involved in cell adhesion. *The EMBO Journal*.

[139] Schäfer M. K. E., Altevogt P. (2010). L1CAM malfunction in the nervous system and human carcinomas. *Cellular and Molecular Life Sciences*.

[140] He Y., Jensen G. J., Bjorkman P. J. (2009). Cryo-electron tomography of homophilic adhesion mediated by the neural cell adhesion molecule L1. *Structure*.

[141] Dahme M., Bartsch U., Martini R., Anliker B., Schachner M., Mantei N. (1997). Disruption of the mouse L1 gene leads to malformations of the nervous system. *Nature Genetics*.

[142] Demyanenko G. P., Tsai A. Y., Maness P. F. (1999). Abnormalities in neuronal process extension, hippocampal development, and the ventricular system of L1 knockout mice. *The Journal of Neuroscience*.

[143] Patzke C., Acuna C., Giam L. R., Wernig M., Südhof T. C. (2016). Conditional deletion of *L1CAM* in human neurons impairs both axonal and dendritic arborization and action potential generation. *The Journal of Experimental Medicine*.

[144] Kajiwara Y., Ueno H., Hashiguchi Y. (2011). Expression of L1 cell adhesion molecule and morphologic features at the invasive front of colorectal cancer. *American Journal of Clinical Pathology*.

[145] Fogel M., Mechtersheimer S., Huszar M. (2003). L1 adhesion molecule (CD 171) in development and progression of human malignant melanoma. *Cancer Letters*.

[146] Shtutman M., Levina E., Ohouo P., Baig M., Roninson I. B. (2006). Cell adhesion molecule L1 disrupts E-cadherin-containing adherens junctions and increases scattering and motility of MCF7 breast carcinoma cells. *Cancer Research*.

[147] Zecchini S., Bianchi M., Colombo N. (2008). The differential role of L1 in ovarian carcinoma and normal ovarian surface epithelium. *Cancer Research*.

[148] Meli M. L., Carrel F., Waibel R. (1999). Anti-neuroblastoma antibody chCE7 binds to an isoform of L1-CAM present in renal carcinoma cells. *International Journal of Cancer*.

[149] Kaifi J. T., Strelow A., Schurr P. G. (2006). L1 (CD171) is highly expressed in gastrointestinal stromal tumors. *Modern Pathology*.

[150] Sebens Müerköster S., Werbing V., Sipos B. (2007). Drug-induced expression of the cellular adhesion molecule L1CAM confers anti-apoptotic protection and chemoresistance in pancreatic ductal adenocarcinoma cells. *Oncogene*.

[151] Hung G., Colton J., Fisher L. (2002). Immunohistochemistry study of human vestibular nerve schwannoma differentiation. *Glia*.

[152] Tsuzuki T., Izumoto S., Ohnishi T., Hiraga S., Arita N., Hayakawa T. (1998). Neural cell adhesion molecule L1 in gliomas: correlation with TGF-beta and p53. *Journal of Clinical Pathology*.

[153] Rawnaq T., Quaas A., Zander H. (2012). L1 is highly expressed in tumors of the nervous system: a study of over 8000 human tissues. *The Journal of Surgical Research*.

[154] Huszar M., Moldenhauer G., Gschwend V., Benarie A., Altevogt P., Fogel M. (2006). Expression profile analysis in multiple human tumors identifies L1 (CD171) as a molecular marker for differential diagnosis and targeted therapy. *Human Pathology*.

[155] Schönmann S. M., Iyer J., Laeng H., Gerber H. A., Käser H., Blaser K. (1986). Production and characterization of monoclonal antibodies against human neuroblastoma. *International Journal of Cancer*.

[156] Figarella-Branger D. F., Durbec P. L., Rougon G. N. (1990). Differential spectrum of expression of neural cell adhesion molecule isoforms and L1 adhesion molecules on human neuroectodermal tumors. *Cancer Research*.

[157] Rached J., Nasr Z., Abdallah J., Abou-Antoun T. (2016). L1-CAM knock-down radiosensitizes neuroblastoma IMR-32 cells by simultaneously decreasing MycN, but increasing PTEN protein expression. *International Journal of Oncology*.

[158] Wachowiak R., Fiegel H. C., Kaifi J. T. (2007). L1 is associated with favorable outcome in neuroblastomas in contrast to adult tumors. *Annals of Surgical Oncology*.

[159] Inaguma S., Wang Z., Lasota J. P., Miettinen M. M. (2016). Expression of neural cell adhesion molecule L1 (CD171) in neuroectodermal and other tumors. An immunohistochemical study of 5155 tumors and critical evaluation of CD171 prognostic value in gastrointestinal stromal tumors. *Oncotarget*.

[160] Amstutz H. P., Rytz C., Novak-Hofer I. (1993). Production and characterization of a mouse human chimeric antibody directed against human neuroblastoma. *International Journal of Cancer*.

[161] Novak-Hofer I., Amstutz H. P., Morgenthaler J. J., Schubiger P. A. (1994). Internalization and degradation of monoclonal antibody chCE7 by human neuroblastoma cells. *International Journal of Cancer*.

[162] Novak-Hofer I., Amstutz H. P., Haldemann A. (1992). Radioimmunolocalization of neuroblastoma xenografts with chimeric antibody chCE7. *Journal of Nuclear Medicine*.

[163] Hoefnagel C. A., Rutgers M., Buitenhuis C. K. M. (2001). A comparison of targetting of neuroblastoma with mIBG and anti L1-CAM antibody mAb chCE7: therapeutic efficacy in a neuroblastoma xenograft model and imaging of neuroblastoma patients. *European Journal of Nuclear Medicine*.

[164] Wang Y., Loers G., Pan H. C. (2012). Antibody fragments directed against different portions of the human neural cell adhesion molecule L1 act as inhibitors or activators of L1 function. *PLoS One*.

[165] Hong H., Stastny M., Brown C. (2014). Diverse solid tumors expressing a restricted epitope of L1-CAM can be targeted by chimeric antigen receptor redirected T lymphocytes. *Journal of Immunotherapy*.

[166] Gonzalez S., Naranjo A., Serrano L. M., Chang W. C., Wright C. L., Jensen M. C. (2004). Genetic engineering of cytolytic T lymphocytes for adoptive T cell therapy of neuroblastoma. *The Journal of Gene Medicine*.

[167] Park J. R., Digiusto D. L., Slovak M. (2007). Adoptive transfer of chimeric antigen receptor re-directed cytolytic T lymphocyte clones in patients with neuroblastoma. *Molecular Therapy*.

[168] Künkele A., Johnson A. J., Rolczynski L. S. (2015). Functional tuning of CARs reveals signaling threshold above which CD8^+^ CTL antitumor potency is attenuated due to cell Fas-FasL-dependent AICD. *Cancer Immunology Research*.

[169] Künkele A., Taraseviciute A., Finn L. S. (2017). Preclinical assessment of CD171-directed CAR T-cell adoptive therapy for childhood neuroblastoma: CE7 epitope target safety and product manufacturing feasibility. *Clinical Cancer Research*.

[170] Hakomori S. (2000). Traveling for the glycosphingolipid path. *Glycoconjugate Journal*.

[171] Ledeen R. W., Wu G. (2002). Ganglioside function in calcium homeostasis and signaling. *Neurochemical Research*.

[172] Lopez P. H. H., Schnaar R. L. (2009). Gangliosides in cell recognition and membrane protein regulation. *Current Opinion in Structural Biology*.

[173] Hakomori S. (2004). Glycosynapses: microdomains controlling carbohydrate-dependent cell adhesion and signaling. *Anais da Academia Brasileira de Ciências*.

[174] Simons K., Ikonen E. (1997). Functional rafts in cell membranes. *Nature*.

[175] Sonnino S., Mauri L., Chigorno V., Prinetti A. (2007). Gangliosides as components of lipid membrane domains. *Glycobiology*.

[176] Molotkovskaya I. M., Kholodenko R. V., Zelenova N. A., Sapozhnikov A. M., Mikhalev I. I., Molotkovsky J. G. (2000). Gangliosides induce cell apoptosis in the cytotoxic line CTLL-2, but not in the promyelocyte leukemia cell line HL-60. *Membrane & Cell Biology*.

[177] Shurin G. V., Gerein V., Lotze M. T., Barksdale E. M. (2000). Apoptosis induced in T cells by human neuroblastoma cells: role of Fas ligand. *Natural Immunity*.

[178] Molotkovskaya I. M., Kholodenko R. V., Molotkovsky J. G. (2002). Influence of gangliosides on the IL-2- and IL-4-dependent cell proliferation. *Neurochemical Research*.

[179] Robbins P. W., Macpherson I. (1971). Control of glycolipid synthesis in a cultured hamster cell line. *Nature*.

[180] Chatterjee S., Sweeley C. C., Velicer L. F. (1975). Glycosphingolipids of human KB cells grown in monolayer, suspension, and synchronized cultures. *The Journal of Biological Chemistry*.

[181] Liour S. S., Kapitonov D., Yu R. K. (2000). Expression of gangliosides in neuronal development of P19 embryonal carcinoma stem cells. *Journal of Neuroscience Research*.

[182] Cheresh D. A., Varki A. P., Varki N. M., Stallcup W. B., Levine J., Reisfeld R. A. (1984). A monoclonal antibody recognizes an O-acylated sialic acid in a human melanoma-associated ganglioside. *The Journal of Biological Chemistry*.

[183] Ledeen R. W., Yu R. K. (1982). [10] Gangliosides: structure, isolation, and analysis. *Methods Enzymol*.

[184] El-Abbadi M., Seyfried T. N., Yates A. J., Orosz C., Lee M. C. (2001). Ganglioside composition and histology of a spontaneous metastatic brain tumour in the VM mouse. *British Journal of Cancer*.

[185] Müthing J., Meisen I., Kniep B. (2005). Tumor-associated CD75s gangliosides and CD75s-bearing glycoproteins with Neu5Ac*α*2-6Gal*β*1-4GlcNAc-residues are receptors for the anticancer drug rViscumin. *The FASEB Journal*.

[186] Kohla G., Stockfleth E., Schauer R. (2002). Gangliosides with O-acetylated sialic acids in tumors of neuroectodermal origin. *Neurochemical Research*.

[187] Wu Z. L., Schwartz E., Seeger R., Ladisch S. (1986). Expression of G_D2_ ganglioside by untreated primary human neuroblastomas. *Cancer Research*.

[188] Schulz G., Cheresh D. A., Varki N. M., Yu A., Staffileno L. K., Reisfeld R. A. (1984). Detection of ganglioside G_D2_ in tumor tissues and sera of neuroblastoma patients. *Cancer Research*.

[189] Portoukalian J., Zwingelstein G., Doré J. F. (1979). Lipid composition of human malignant melanoma tumors at various levels of malignant growth. *European Journal of Biochemistry*.

[190] Schengrund C. L., Shochat S. J. (1988). Gangliosides in neuroblastomas. *Neurochemical Pathology*.

[191] Tsuchida T., Saxton R. E., Morton D. L., Irie R. F. (1987). Gangliosides of human melanoma. *Journal of the National Cancer Institute*.

[192] Kaucic K., Etue N., LaFleur B., Woods W., Ladisch S. (2001). Neuroblastomas of infancy exhibit a characteristic ganglioside pattern. *Cancer*.

[193] Schengrund C. L., Repman M. A., Shochat S. J. (1985). Ganglioside composition of human neuroblastomas. Correlation with prognosis. A Pediatric Oncology Group Study. *Cancer*.

[194] Hettmer S., Malott C., Woods W., Ladisch S., Kaucic K. (2003). Biological stratification of human neuroblastoma by complex ‘B’ pathway ganglioside expression. *Cancer Research*.

[195] Dong L., Liu Y., Colberg-Poley A. M., Kaucic K., Ladisch S. (2011). Induction of GM1a/GD1b synthase triggers complex ganglioside expression and alters neuroblastoma cell behavior; a new tumor cell model of ganglioside function. *Glycoconjugate Journal*.

[196] Hettmer S., McCarter R., Ladisch S., Kaucic K. (2004). Alterations in neuroblastoma ganglioside synthesis by induction of GD1b synthase by retinoic acid. *British Journal of Cancer*.

[197] Hakomori S. (1996). Tumor malignancy defined by aberrant glycosylation and sphingo(glyco)-lipid metabolism. *Cancer Research*.

[198] Ladisch S., Wu Z. L., Feig S. (1987). Shedding of G_D2_ ganglioside by human neuroblastoma. *International Journal of Cancer*.

[199] Ladisch S., Gillard B., Wong C., Ulsh L. (1983). Shedding and immunoregulatory activity of YAC-1 lymphoma cell gangliosides. *Cancer Research*.

[200] Bernhard H., Zum Büschenfelde K. H. M., Dippold W. G. (1989). Ganglioside GD3 shedding by human malignant melanoma cells. *International Journal of Cancer*.

[201] Merritt W. D., Der-Minassian V., Reaman G. H. (1994). Increased GD3 ganglioside in plasma of children with T-cell acute lymphoblastic leukemia. *Leukemia*.

[202] Chang F., Li R., Ladisch S. (1997). Shedding of gangliosides by human medulloblastoma cells. *Experimental Cell Research*.

[203] Ladisch S., Li R., Olson E. (1994). Ceramide structure predicts tumor ganglioside immunosuppressive activity. *Proceedings of the National Academy of Sciences of the United States of America*.

[204] Li R., Villacreses N., Ladisch S. (1995). Human tumor gangliosides inhibit murine immune responses *in vivo*. *Cancer Research*.

[205] Ziegler-Heitbrock H. W., Kafferlein E., Haas J. G. (1992). Gangliosides suppress tumor necrosis factor production in human monocytes. *Journal of Immunology*.

[206] Ladisch S., Becker H., Ulsh L. (1992). Immunosuppression by human gangliosides: I. Relationship of carbohydrate structure to the inhibition of T cell responses. *Biochimica et Biophysica Acta (BBA) - Lipids and Lipid Metabolism*.

[207] Chu J. W., Sharom F. J. (1995). Gangliosides interact with interleukin-4 and inhibit interleukin-4-stimulated helper T-cell proliferation. *Immunology*.

[208] Li R., Gage D., McKallip R., Ladisch S. (1996). Structural characterization and *in vivo* immunosuppressive activity of neuroblastoma G_D2_. *Glycoconjugate Journal*.

[209] Ladisch S., Kitada S., Hays E. F. (1987). Gangliosides shed by tumor cells enhance tumor formation in mice. *The Journal of Clinical Investigation*.

[210] Valentino L., Moss T., Olson E., Wang H. J., Elashoff R., Ladisch S. (1990). Shed tumor gangliosides and progression of human neuroblastoma. *Blood*.

[211] Lammie G., Cheung N., Gerald W., Rosenblum M., Cordoncardo C. (1993). Ganglioside GD(2) expression in the human nervous-system and in neuroblastomas - an immunohistochemical study. *International Journal of Oncology*.

[212] Svennerholm L., Boström K., Fredman P. (1994). Gangliosides and allied glycosphingolipids in human peripheral nerve and spinal cord. *Biochimica et Biophysica Acta (BBA) - Lipids and Lipid Metabolism*.

[213] Martinez C., Hofmann T. J., Marino R., Dominici M., Horwitz E. M. (2007). Human bone marrow mesenchymal stromal cells express the neural ganglioside GD2: a novel surface marker for the identification of MSCs. *Blood*.

[214] Terzic T., Cordeau M., Herblot S. (2017). Expression of disialoganglioside (GD2) in neuroblastic tumors: a prognostic value for patients treated with anti-GD2 immunotherapy. *Pediatric and Developmental Pathology*.

[215] Cheresh D. A., Pierschbacher M. D., Herzig M. A., Mujoo K. (1986). Disialogangliosides GD2 and GD3 are involved in the attachment of human melanoma and neuroblastoma cells to extracellular matrix proteins. *The Journal of Cell Biology*.

[216] Zeng Y., Fest S., Kunert R. (2005). Anti-neuroblastoma effect of ch14.18 antibody produced in CHO cells is mediated by NK-cells in mice. *Molecular Immunology*.

[217] Mora J. (2016). Dinutuximab for the treatment of pediatric patients with high-risk neuroblastoma. *Expert Review of Clinical Pharmacology*.

[218] Sait S., Modak S. (2017). Anti-GD2 immunotherapy for neuroblastoma. *Expert Review of Anticancer Therapy*.

[219] Horta Z. P., Goldberg J. L., Sondel P. M. (2016). Anti-GD2 mAbs and next-generation mAb-based agents for cancer therapy. *Immunotherapy*.

[220] Suzuki M., Cheung N. K. V. (2014). Disialoganglioside GD2 as a therapeutic target for human diseases. *Expert Opinion on Therapeutic Targets*.

[221] Yu A. L., Uttenreuther-Fischer M. M., Huang C. S. (1998). Phase I trial of a human-mouse chimeric anti-disialoganglioside monoclonal antibody ch14.18 in patients with refractory neuroblastoma and osteosarcoma. *Oncologia*.

[222] Cheung N. K. V., Guo H., Hu J., Tassev D. V., Cheung I. Y. (2014). Humanizing murine IgG3 anti-GD2 antibody m3F8 substantially improves antibody-dependent cell-mediated cytotoxicity while retaining targeting in vivo. *OncoImmunology*.

[223] Navid F., Sondel P. M., Barfield R. (2014). Phase I trial of a novel anti-GD2 monoclonal antibody, Hu14.18K322A, designed to decrease toxicity in children with refractory or recurrent neuroblastoma. *Oncologia*.

[224] Sorkin L. S., Otto M., Baldwin W. M. (2010). Anti-GD2 with an FC point mutation reduces complement fixation and decreases antibody-induced allodynia. *Pain*.

[225] Sjoberg E. R., Manzi A. E., Khoo K. H., Dell A., Varki A. (1992). Structural and immunological characterization of O-acetylated GD2. Evidence that GD2 is an acceptor for ganglioside O-acetyltransferase in human melanoma cells. *The Journal of Biological Chemistry*.

[226] Fleurence J., Fougeray S., Bahri M. (2017). Targeting *O*-acetyl-GD2 ganglioside for cancer immunotherapy. *Journal of Immunology Research*.

[227] Alvarez-Rueda N., Desselle A., Cochonneau D. (2011). A monoclonal antibody to O-acetyl-GD2 ganglioside and not to GD2 shows potent anti-tumor activity without peripheral nervous system cross-reactivity. *PLoS One*.

[228] Terme M., Dorvillius M., Cochonneau D. (2014). Chimeric antibody c.8B6 to O-acetyl-GD2 mediates the same efficient anti-neuroblastoma effects as therapeutic ch14.18 antibody to GD2 without antibody induced allodynia. *PLoS One*.

[229] Yoshida S., Fukumoto S., Kawaguchi H., Sato S., Ueda R., Furukawa K. (2001). Ganglioside G_D2_ in small cell lung cancer cell lines: enhancement of cell proliferation and mediation of apoptosis. *Cancer Research*.

[230] Kowalczyk A., Gil M., Horwacik I., Odrowaz Z., Kozbor D., Rokita H. (2009). The GD2-specific 14G2a monoclonal antibody induces apoptosis and enhances cytotoxicity of chemotherapeutic drugs in IMR-32 human neuroblastoma cells. *Cancer Letters*.

[231] Doronin I. I., Vishnyakova P. A., Kholodenko I. V. (2014). Ganglioside GD2 in reception and transduction of cell death signal in tumor cells. *BMC Cancer*.

[232] Zhu W., Mao X., Wang W. (2018). Anti-ganglioside GD2 monoclonal antibody synergizes with cisplatin to induce endoplasmic reticulum-associated apoptosis in osteosarcoma cells. *Pharmazie*.

[233] Faraj S., Bahri M., Fougeray S. (2017). Neuroblastoma chemotherapy can be augmented by immunotargeting O-acetyl-GD2 tumor-associated ganglioside. *OncoImmunology*.

[234] Brown B. S., Patanam T., Mobli K. (2014). Etoposide-loaded immunoliposomes as active targeting agents for GD2-positive malignancies. *Cancer Biology & Therapy*.

[235] Brignole C., Pastorino F., Marimpietri D. (2004). Immune cell-mediated antitumor activities of GD2-targeted liposomal c-myb antisense oligonucleotides containing CpG motifs. *Journal of the National Cancer Institute*.

[236] Di Paolo D., Ambrogio C., Pastorino F. (2011). Selective therapeutic targeting of the anaplastic lymphoma kinase with liposomal siRNA induces apoptosis and inhibits angiogenesis in neuroblastoma. *Molecular Therapy*.

[237] Baiu D. C., Artz N. S., McElreath M. R. (2015). High specificity targeting and detection of human neuroblastoma using multifunctional anti-GD2 iron-oxide nanoparticles. *Nanomedicine*.

[238] Tivnan A., Orr W. S., Gubala V. (2012). Inhibition of neuroblastoma tumor growth by targeted delivery of microRNA-34a using anti-disialoganglioside GD2 coated nanoparticles. *PLoS One*.

[239] Pastorino F., Brignole C., Loi M. (2013). Nanocarrier-mediated targeting of tumor and tumor vascular cells improves uptake and penetration of drugs into neuroblastoma. *Frontiers in Oncology*.

[240] Zubareva A. A., Boyko A. A., Kholodenko I. V. (2016). Chitosan nanoparticles targeted to the tumor-associated ganglioside GD2. *Russian Journal of Bioorganic Chemistry*.

[241] Zeng Y., Huebener N., Fest S. (2007). Fractalkine (CX3CL1)- and interleukin-2-enriched neuroblastoma microenvironment induces eradication of metastases mediated by T cells and natural killer cells. *Cancer Research*.

[242] Otto M., Barfield R. C., Martin W. J. (2005). Combination immunotherapy with clinical-scale enriched human *γδ* T cells, hu14.18 antibody, and the immunocytokine Fc-IL7 in disseminated neuroblastoma. *Clinical Cancer Research*.

[243] Wargalla U. C., Reisfeld R. A. (1989). Rate of internalization of an immunotoxin correlates with cytotoxic activity against human tumor cells. *Proceedings of the National Academy of Sciences of the United States of America*.

[244] Mujoo K., Reisfeld R. A., Cheung L., Rosenblum M. G. (1991). A potent and specific immunotoxin for tumor cells expressing disialoganglioside GD2. *Cancer Immunology, Immunotherapy*.

[245] Thomas P. B., Delatte S. J., Sutphin A., Frankel A. E., Tagge E. P. (2002). Effective targeted cytotoxicity of neuroblastoma cells. *Journal of Pediatric Surgery*.

[246] Tur M. K., Sasse S., Stöcker M. (2001). An anti-GD2 single chain Fv selected by phage display and fused to Pseudomonas exotoxin A develops specific cytotoxic activity against neuroblastoma derived cell lines. *International Journal of Molecular Medicine*.

[247] Modak S., Cheung N. K. (2005). Antibody-based targeted radiation to pediatric tumors. *Journal of Nuclear Medicine*.

[248] Kholodenko R. V., Kalinovsky D. V., Doronin I. I., Ponomarev E. D., Kholodenko I. V. (2017). Antibody fragments as potential biopharmaceuticals for cancer therapy: success and limitations. *Current Medicinal Chemistry*.

[249] Pastorino F., Brignole C., Marimpietri D. (2003). Doxorubicin-loaded Fab’ fragments of anti-disialoganglioside immunoliposomes selectively inhibit the growth and dissemination of human neuroblastoma in nude mice. *Cancer Research*.

[250] Cheung N. K., Modak S., Lin Y. (2004). Single-chain Fv-streptavidin substantially improved therapeutic index in multistep targeting directed at disialoganglioside GD2. *Journal of Nuclear Medicine*.

[251] Sedykh S. E., Prinz V. V., Buneva V. N., Nevinsky G. A. (2018). Bispecific antibodies: design, therapy, perspectives. *Drug Design, Development and Therapy*.

[252] Deyev S. M., Lebedenko E. N. (2017). Targeted bifunctional proteins and hybrid nanoconstructs for cancer diagnostics and therapies. *Molekuliarnaia Biologiia*.

[253] Deppisch N., Ruf P., Eissler N. (2015). Efficacy and tolerability of a GD2-directed trifunctional bispecific antibody in a preclinical model: subcutaneous administration is superior to intravenous delivery. *Molecular Cancer Therapeutics*.

[254] Cheng M., Santich B. H., Xu H., Ahmed M., Huse M., Cheung N. K. V. (2016). Successful engineering of a highly potent single-chain variable-fragment (scFv) bispecific antibody to target disialoganglioside (GD2) positive tumors. *OncoImmunology*.

[255] Xu H., Cheng M., Guo H., Chen Y., Huse M., Cheung N. K. V. (2015). Retargeting T cells to GD2 pentasaccharide on human tumors using bispecific humanized antibody. *Cancer Immunology Research*.

[256] Hoseini S. S., Dobrenkov K., Pankov D., Xu X. L., Cheung N. K. V. (2017). Bispecific antibody does not induce T-cell death mediated by chimeric antigen receptor against disialoganglioside GD2. *OncoImmunology*.

[257] Bernards R., Dessain S. K., Weinberg R. A. (1986). N-myc amplification causes down-modulation of MHC class I antigen expression in neuroblastoma. *Cell*.

[258] Pulè M. A., Straathof K. C., Dotti G., Heslop H. E., Rooney C. M., Brenner M. K. (2005). A chimeric T cell antigen receptor that augments cytokine release and supports clonal expansion of primary human T cells. *Molecular Therapy*.

[259] Yvon E., Del Vecchio M., Savoldo B. (2009). Immunotherapy of metastatic melanoma using genetically engineered GD2-specific T cells. *Clinical Cancer Research*.

[260] Louis C. U., Savoldo B., Dotti G. (2011). Antitumor activity and long-term fate of chimeric antigen receptor-positive T cells in patients with neuroblastoma. *Blood*.

[261] Pule M. A., Savoldo B., Myers G. D. (2008). Virus-specific T cells engineered to coexpress tumor-specific receptors: persistence and antitumor activity in individuals with neuroblastoma. *Nature Medicine*.

[262] Yong C. S. M., Dardalhon V., Devaud C., Taylor N., Darcy P. K., Kershaw M. H. (2017). CAR T-cell therapy of solid tumors. *Immunology and Cell Biology*.

[263] Xu X., Qiu J., Sun Y. (2017). The basics of CAR T design and challenges in immunotherapy of solid tumors - ovarian cancer as a model. *Human Vaccines & Immunotherapeutics*.

[264] Long A. H., Haso W. M., Shern J. F. (2015). 4-1BB costimulation ameliorates T cell exhaustion induced by tonic signaling of chimeric antigen receptors. *Nature Medicine*.

[265] Muhammad N., Mao Q., Xia H. (2017). CAR T-cells for cancer therapy. *Biotechnology & Genetic Engineering Reviews*.

[266] Hombach A., Hombach A. A., Abken H. (2010). Adoptive immunotherapy with genetically engineered T cells: modification of the IgG1 Fc ‘spacer’ domain in the extracellular moiety of chimeric antigen receptors avoids ‘off-target’ activation and unintended initiation of an innate immune response. *Gene Therapy*.

[267] Sun J., Dotti G., Huye L. E. (2010). T cells expressing constitutively active Akt resist multiple tumor-associated inhibitory mechanisms. *Molecular Therapy*.

[268] Gargett T., Yu W., Dotti G. (2016). GD2-specific CAR T cells undergo potent activation and deletion following antigen encounter but can be protected from activation-induced cell death by PD-1 blockade. *Molecular Therapy*.

[269] Heczey A., Louis C. U. (2013). Advances in chimeric antigen receptor immunotherapy for neuroblastoma. *Discovery Medicine*.

[270] Heczey A., Louis C. U., Savoldo B. (2017). CAR T cells administered in combination with lymphodepletion and PD-1 inhibition to patients with neuroblastoma. *Molecular Therapy*.

